# An online survey of women’s views of respectful and disrespectful pregnancy and early labour care in the Czech Republic

**DOI:** 10.1186/s12884-024-06448-5

**Published:** 2024-05-15

**Authors:** Deirdre Daly, Natalie Sedlicka, Kateřina Švanderlíková, PetraAnn Ann Kovařčíková, Radka Wilhelmová, Cecily Begley

**Affiliations:** 1 School of Nursing and Midwifery, Trinity College Dublin, 24 D’Olier Street, Dublin, DO2 T283 Ireland; 2 Association for Birth Houses & Centers (APODAC), Týnská ulička 1064/6, , Prague 1, 11000 Czech Republic; 3 Faculty of Medicine, Department of Health Sciences, Masaryk University, Brno, Czech Republic

**Keywords:** Consent, Decision-making, Maternity care, Respect, Disrespect, Survey

## Abstract

**Objective:**

To ascertain and explore the views of women and their partners, giving birth in the Czech Republic, of the level of respectful or disrespectful care provided during pregnancy and early labour.

**Design:**

Ethical approval was granted for a descriptive, online anonymous survey of 65 questions, with quantitative and qualitative responses.

**Setting:**

The Czech Republic.The survey was completed by 8,767 women and 69 partners in 2018.

**Measurements and findings:**

Descriptive statistics and thematic analysis were used to present results. The majority of women were aged 26-35 years. Most had birthed in one of 93 hospitals, with 1.5% home births. Almost 40% never had an abdominal examination.in pregnancy. Quantitative data analysis revealed that less than half were given information on place of birth, or how to keep labour normal or non-interventionist. Almost 60% did not get information on positions for birth. Most (68%) commenced labour naturally, 25% had labour induced, 40% of them before term, and 7% had an elective caesarean section; 55% stated they had not been given any choice in the decision. Over half of those who had a membrane sweep said permission had not been sought. Half (54%) only had ‘checking’ visits from the midwife in labour.

**Key conclusions:**

Findings reveal a lack of information-giving, discussion and shared decision-making from healthcare professionals during pregnancy and early labour. Some practices were non-evidenced-based, and interventions were sometimes made without consent.

**Implications for practice:**

The examples of disrespectful care described in this study caused women distress during childbirth, which may result in an increased fear of childbirth or an increase in free-birthing.

**Supplementary Information:**

The online version contains supplementary material available at 10.1186/s12884-024-06448-5.

## Background

The Czech Republic became a separate state in 1993, after 40 years of socialism and communism in Czechoslovakia ended in 1989. Under communism, the rights of the whole population were repressed and their freedom was restricted. Women, in particular, had little autonomy in what was a very patriarchal country. Since 1993, restrictions have been reduced and women are now more independent and autonomous. However, in the maternity care services, little has changed and the model of care is still one of hierarchy and paternalism, where midwives are not recognised or respected [1]. Midwifery as a profession was eliminated by the former regime and its reintroduction is a slow process. The professionalisation and the professional authority of midwives is part of the debate on structural health care system change in the context of resistance to giving up the dominant position of Czech gynaecologists and obstetricians in delivery rooms [2]. In many hospitals, normal births are conducted by obstetricians, with midwives assisting. Midwives are suppressed [2], not permitted to work autonomously, or to attend home births [3] and a recent Ministry of Health publication on perinatal care does not mention midwives as core care providers [4]. In their book, Games of Life, Šmídová et al. (2015) discuss biomedicine’s approach to childbirth in the Czech Republic as being as a state of emergency and high risk and dealt with as a highly medicalised event using routine interventions as precautions [2]. Although the Czech Republic joined the EU in 2004, midwives are precluded from using their full range of competencies or from offering private independent care [3]. In addition, the cost of midwifery care to women is not covered by the standard public health insurance and some women opt to give birth at home, either alone or with the help of a doula [3]. Set in the context of improving the quality of maternity care, the Ministry of Health sought ‘Experiences of Obstetrics’ from parents via their Facebook page in 2016. A total of 689 contributions from 486 original comments were posted and analysed to identify recommendations. Overall, the analysis showed clear requirements for changing the system which the authors described as medically-guided childbirth and care with a high level of intervention. The authors concluded that practically all proposed recommendations were aimed at enabling non-interventional births. Other recommendations referred to the choice of place of birth and using of the latest scientific evidence to inform practice [5]. In addition, some maternity hospitals have introduced radically different regimens of care and embraced practises such as gentle birthing, acupuncture, fathers in labour rooms, and other innovations that aimed humanise childbirth [6].

There were 93 maternity hospitals in the Czech Republic in 2018. Since 2019, pilot Centres of Midwifery (CPA) were opened. First, two birth apartments were built in Prague with midwives starting to provide prenatal care for women with no, or low, medical and obstetric risks, in addition to additional obstetric care provided elsewhere. However, an obstetrician must decide if the midwife can be the primary carer during birth. The second CPA opened in Brno in 2021, with midwives supporting and leading physiological birth. It is important to acknowledge that two midwifery centres existed in the early 2000s but, despite having favourable birth outcomes, the second of these was closed in 2007 (http://www.biostatisticka.cz/jak-to-byvalo-ve-vrchlabi/).

Respectful maternity care is a human right [7]. In 2014, the World Health Organization (WHO) re-emphasised that being disrespected in childbirth not only infringed women’s rights but also discouraged women from using maternity care services [8], which could adversely impact on their health [9]. Disrespectful care can comprise physical abuse, discrimination, abandonment, or detention in facilities but also care that is non-consented, non-confidential, or non-dignified [10]. Miller et al. [11] associated evidence-based care with respectful care indicating that care that is non-evidence based is un-dignified, and disrespects women’s rights. More recently Lappeman and Swartz [12] suggested that silence between healthcare providers and the women in their care also can be a form of neglect. Findings from systematic reviews [13, 14] and numerous empirical quantitative and qualitative studies demonstrate its widespread existence across the world [15–19]. Disrespectful care during childbirth is not a new phenomenon and, by exploring the long history of obstetric violence, O’Brien and Rich [20] locate biological reproduction as a site of social violence.

In the context of the post-socialist transformation in the Czech Republic, many women’s maternity care needs are still not paramount and many have no autonomy [1, 3], shown by healthcare professionals not preserving women’s dignity or privacy during examinations or when in labour and, sometimes, ignoring women’s refusal of consent or interventions [1, 21].

The Czech Republic does not publish complete data on maternity care practices, intervention rates or statistics either in individual hospitals, or on births at home. In 2017, a private statistician started a court case to seek access to data on care in all maternity hospitals, but her request has not yet been granted [22].

This paper presents results from an anonymous online survey of women’s, or their partners, views on the care experienced when pregnant with and giving birth to their first baby in the Czech Republic. It follows a similar study of healthcare professionals’ views [23], conducted following a television documentary shown in the Czech Republic that appeared to indicate the use of some poor practices in maternity care [21]. A later documentary, which included women’s narratives, also highlighted the continuing existence of malpractices and lack of evidence-based practices [24]. Apart from these documentaries, and anecdotal evidence, there are minimal data on women’s or couples’ experiences of maternity care and services in the Czech Republic.

## Methods

The study aimed to ascertain and explore the views of women, and partners of women, who had given birth in the Czech Republic of the level of respectful or disrespectful care provided for them during pregnancy and early labour. A descriptive online anonymous survey containing 65 questions was designed (Additional file S1). Five questions related to respondents and the age of their child(ren). Thirteen questions related to specific antenatal and intrapartum actions/interventions (e.g., induction of labour, application of electronic fetal monitoring etc.) had ‘Yes/No/Not applicable’ responses and 47 questions included open text comments to enable respondents to elaborate on the care, intervention(s) experienced or interactions with healthcare professionals. The quantitative questions were based on evidence and recommendations from relevant Cochrane reviews which were used in a previous survey of maternity care professionals’ views of respectful and disrespectful maternity care in the Czech Republic [23]. We also incorporated questions relating to nine of the 12 domains identified in a review of 67 studies from 32 countries on women’s perspectives of respectful maternity care [25]. These included: being free from harm and mistreatment, maintaining privacy and confidentiality, preserving women’s dignity, prospective provision of information and seeking informed consent, ensuring continuous access to family and community support, engaging with effective communication, respecting women’s choices that strengthens their capabilities to give birth, and provision of efficient and effective care and continuity of care. We did not include questions on the physical environment or resources, equity of services or competence and motivations of personnel.

The survey content was developed with nine midwives, doulas and women who had birthed in the Czech Republic, and assessed for acceptability and face validity with 20 women. The survey was prepared in English by CB and DD and translated into the Czech language, and back-translated from Czech to English by NS, KS, PK. Consistency was also checked between the two versions by two other bilingual volunteers. Ethical approval was granted by the Research Ethics Committee, School of Nursing and Midwifery, Trinity College Dublin [14 November 2016].

## Participants

Participants were women aged 18 years and over, or partners of women, who had given birth in the Czech Republic. All participants were informed about the study and given the opportunity to ask questions. All were asked to complete the survey in relation to their experiences while pregnant and giving birth to their first baby.

## Recruitment

We identified volunteer representatives in each region, through national professional organisations, universities, midwifery schools, hospitals, other health/helping professional fields, participants at birthing seminars (APODAC, UNIPA, etc.), who advertised and provided information on the study in local places/services such as maternity centres, kindergartens, lactation advisors, doulas, associations concerned about birth topics or child education, national magazines and other media. The survey was mainly distributed through personal recruitment (emailing/facebook notices, messages and sharing), with women sharing it with others thereafter. The main channel was Facebook (APODAC pages, JakJinak and other personal profiles of the members of the association) but the survey was then also advertised by various maternal centres and mother and baby websites, and other professional organisations. Those willing to take part completed the survey online, between 1st March 2018 and 31st May 2018. At the start of the survey, participants were informed that the survey was anonymous and that submission of the survey was taken as giving consent to participate.

## Data collection

The survey was anonymous, prepared in the Czech language, and administered via Survey Monkey®.

Participants were asked;

(i) their age range, how old their youngest and oldest children were, the name of the hospital they gave birth in (or if they had a home birth);
(ii) to answer all questions in relation to their first-born baby;
(iii) what information they were given on aspects such as: choices available for place of birth, mode of birth or how to keep labour remaining natural or non-interventionist;
(iv) whether or not they had abdominal examinations performed in pregnancy;
(v) whether or not they had, and whether or not they were offered choice regarding induction of labour or elective caesarean section.

## Data analysis

Quantitative data were analysed using descriptive statistics, and the frequency with which care practices or interventions occurred are presented as proportions. Respondents’ open text comments were analysed by a team of four researchers applying descriptive codes, merging codes under category headings, followed by thematic analysis using a data-driven approach [26]. For example, comments relating to consenting to/declining an intervention were categorised as ‘Consent’, ‘Refusal’, or ‘Healthcare practitioners’ reaction’ when care was declined, etc., and comments relating to induction of labour were categorised as ‘Reasons given for induction’, ‘Reasons given for timing of induction’, etc. The team worked together initially to develop code definitions, and then independently, with a final phase of consensus-seeking to ensure comparability across all codes and categories. The first and last authors then acted as peer debriefers [27], adjudicating on the appropriateness of the thematic analysis, once translated into English. When appropriate, direct quotations (translated, as necessary, into English), are used to complement the quantitative data.

## Results

### Characteristics of the sample

A total of 10,200 people completed part of the survey, and 8,920 completed the full survey. Data from one respondent aged less than 18 years was removed, under the terms of our ethical approval. Of the remaining sample, 8,767 (98.31%) were women who had had a baby in the Czech Republic, and 69 (0.77%) were partners of women who had birthed in the Czech Republic and were reporting in relation to their partner’s experiences. The remainder were ‘another person interested in maternity care’ (*n* = 23), a midwife (*n* = 43), a doula (*n* = 6), and ‘other healthcare worker providing maternity care in the Czech Republic’ (*n* = 11). Although all participants had been asked (if they were a healthcare professional) to answer about their own experiences, for the purpose of this arm of the study, these participants’ responses (*n* = 83, 0.92%) were omitted, as we had published healthcare professionals’ views already [23]. This left a final sample size of 8,836. For half the participants, their first baby’s birth had taken place either within the month prior to completing the survey (5.25%, *n* = 462) or between one month to one year previously (43.70%, *n* = 3,846), with the remaining participants (51.05%, *n* = 4,493) completing the survey more than one year after the first birth.

Responses to questions varied according to question type and place in the survey. For example, response rates for the first 21 questions varied from 91% to 100%, while those for the more detailed questions on labour and birth (questions 22–35) varied from 80% to 89%. Later questions (36 to 65) had response rates from 76% to 80%, with question 39 (‘Were you allowed to eat light diet in labour?’) gaining the lowest response rate of 73%. In all questions, results are given as percentages of the actual number responding.

The majority of those who gave their age (*n* = 8,817) were between 26 and 30 years old (*n* = 3,258, 36.95%), with a further 33.33% (*n* = 2,939) aged 31–35 years (Table 1). The average age was 30 years.

**Table 1:** Age of respondents

Age	Number	Percentage
18–25	1,251	14.19
26–30	3,258	36.95
31–35	2,939	33.33
36–40	1,030	11.68
Over 40	339	3.85
Total	8,817	100

Half of the respondents had just one child (*n* = 4,428, 50.23%) and the remainder had other children aged one to four years (*n* = 2,500, 28.36%), five to eight years (*n* = 1,023, 11.60%) or more than 8 years of age (*n* = 864, 9.80%). Respondents were asked where they, or their partner, had given birth. A small number (*n* = 132, 1.50%) had given birth at home. The remainder had birthed in one of 93 hospitals (Additional file S2).

The quantitative findings on experiences are presented in tables and complemented with illustrative direct quotes from respondents’ free-text comments. The proportion of comments are also given, when available, to show the frequency of their use.

### Choice and care during pregnancy

Care in pregnancy included the obstetrician/gynaecologist, staff in the hospital (e.g., hospital midwife, nurse, other health care professionals – postnatal nurse, doctor etc., or private midwife permitted to provide care in hospital but as a doula only, or doula), or the woman’s ‘secret home-birth midwife’ performing an abdominal examination once or twice (*n* = 2,227, 25.53%), on every visit (*n* = 3,058, 35.06%) or never (*n* = 3,438, 39.41%). Less than half of the women, when attending for care in their first pregnancy, were given information by staff caring for them on where or how they might give birth, and only a quarter were given information on how they might keep labour natural or avoid interventions (Table 2).

**Table 2:** Information given to women by their obstetrician, staff in the hospital, or their (home birth) midwife or doula

Type of information	Yes n (%)	No n (%)	Cannot remember n (%)	Total
Choices available for place of birth	4,263 (48.56)	3,905 (44.48)	611 (6.96)	8,779
Choices available for mode of birth	3,624 (41.29)	4,798 (54.67)	355 (4.04)	8,777
Positions you could use for labour and birth	3,263 (37.22)	5,212 (59.46)	291 (3.32)	8,766
The benefit and importance of labour remaining natural or non-interventionist	2,766 (31.55)	5,507 (62.82)	494 (5.63)	8,767
How to keep labour remaining natural or non-interventionist	2,221 (25.34)	5,906 (67.38)	638 (7.28)	8,765

### Mode of birth and gestation at birth

Table 3 shows mode of birth, method of induction of labour and gestation at birth. For the majority of women in their first pregnancy, labour started spontaneously at, or after, their due date (*n* = 5,652, 63.96%). The overall induction of labour rate was 25.03% (*n* = 2,077/8,299) and the overall elective CS rate was 7.04% (*n* = 584/8,299). Half of all women who had labour induced said this occurred before term (*n* = 944, 38.16%) or at term (*n* = 282, 11.40%). Of those who had a CS, 835 women (49.29%) said this was performed before term, and 195 (11.51%) at term, with a further 21.84% (*n* = 370) having their elective CS by 41 weeks’ gestation.

**Table 3:** Mode of birth and gestation at birth

Mode of birth	Spontaneous *n* = 5,652 (63.96%)	Induced *n* = 2,077 (77.36%)	Elective caesarean section *n* = 584 (21.75%)
Gestation at birth*			
Before term		944 (38.16%)	835 (49.29%)
Term	5,652 (63.96%)	282 (11.40%)	195 (11.51%)
1–2 days post-term		213 (8.61%)	370 (21.84%)
3–4 days post-term		148 (5.98%)	
5–6 days post-term		138 (5.58%)	
41 weeks		128 (5.17%)	
Method of induction of labour*			
Sweeping the membranes during a vaginal examination	-	*n* = 1,364 (35.43%)	-
Rupturing membranes	-	*n* = 1,881 (48.86%)	-
A drug vaginally	-	*n* = 1,786 (46.39%)	-
A drug intravenously	-	*n* = 868 (22.55%)	-

*Numbers and proportions vary because not all respondents completed each question

The majority (*n* = 2,543, 94.71%) gave qualitative comments on why they or their carers had not waited for labour to start spontaneously. The reasons for inducing labour included, mainly, *‘pregnancy after term*’ (*n* = 709, 27.88%), which had been explained to some women as being between 40 weeks and 40 weeks and 4 days to 41 weeks and 6 days. Very few women stated ‘after 42 weeks’ (*n* = 14, 0.55%), and 191 women (7.51%) stated ‘before term’ without adding comments. Some women (*n* = 126) said that they were informed by their doctors that it was illegal to permit pregnancy to go far over-due (sometimes as little as 40 weeks plus one day); for example, one woman said that her doctor had said: ‘C*zech law does not allow us to wait more than 41 weeks and 3 days’*. Comments made by the women as to how ‘post-term’ had been described to them included: *‘Everything over 41 weeks may be dangerous for the baby, there is no more time for waiting*’; ‘*You are already a couple of days after term so we make it a bit quicker’; ‘You are overdue, it’s already after 38th week’*, or ‘*From week 40 it is post-term so we have to induce it’.* One woman said: ‘*Doctor was afraid of overdue. That’s why she did Hamilton manoeuvre the 9th of March, the little one was born 10th. My expected date of birth was 17th’.*

The second most common reason for induction was *‘Medical reasons due to maternal factors’* (*n* = 583, 22.93%) (e.g., pre-eclampsia) and ‘*Medical reasons due to baby factors*’ was third (*n* = 332, 13.06%). This included ‘*baby is too big*’ (*n* = 167); ‘*position of the baby*’ (*n* = 140 (92 women were induced because of fetal breech presentation and 29 of those were born by CS, 14 with fetal breech presentation as the primary reason for CS)); ‘*bad fetal heart tracing*’ (*n* = 109) or ‘*baby is not growing*’ (*n* = 46, of whom 11 had actual growth restriction diagnosed). Women often gave a combination of reasons therefore percentages are not given. One woman commented that ‘*the doctor said the placenta could be old already but I was not at term yet and after birth the midwife said it was perfectly ok’*, and a further 309 women (14.88%) stated that no reason was given for induction.

Women who had had an induced labour or a CS were asked if they felt the reason why this was necessary was discussed sufficiently with them (including all the positives and negatives). Just under half (*n* = 1,624, 49.11%) thought that it had been discussed sufficiently and the remainder (*n* = 1,683, 50.89%) did not. Less than half of women (*n* = 1,472, 45.14%) felt they had been offered choice in the decision but 1,789 women (54.86%) stated that they had no choice. The majority had labour induced by rupturing the membranes, with or without use of drugs (Table 3). Of those who had a membrane sweep performed, 908 (56.33%) said the doctor had not asked for their permission before doing this.

### Care provider and support during labour and birth

The quantitative data showed that women were usually cared for in labour with their first baby by an obstetrician/gynaecologist or doctor (*n* = 6,778, 76.88%), and/or a hospital midwife (*n* = 6,626, 75.16%). Small numbers of women were cared for by a private midwife who had a contract with the hospital (and was permitted to care for them in labour as a midwife) (*n* = 212, 2.41%), or a private midwife without a contract with the hospital (who was permitted to care for them in labour as a doula only) (*n* = 197, 2.24%). Doulas cared for 246 women (2.79%), and 388 (4.40%) were cared for by ‘other people’. No additional comments on ‘others’ were provided. Ninety-seven women (1.10%) were cared for by an obstetrician/gynaecologist or doctor who was given an extra payment directly by them.

A small minority of women (*n* = 615, 7.57%) were not accompanied by any lay person in labour. The majority were supported by the baby’s father (*n* = 6,617, 81.32%), a private midwife (*n* = 276, 3.39%), doula (*n* = 242, 2.97%), or another person (*n* = 385, 4.73%). In general, these companions were allowed to stay with them (pre-Covid pandemic) for the whole of their labour (with short breaks) (*n* = 5,911, 82.05%) or for about half the labour (*n* = 502, 6.87%); however, 11% of women were only permitted to have their companions for a short time at the start, or end, of labour (*n* = 475, 6.50%) or not at all (*n* = 316, 4.35%). Almost three-quarters of the women (*n* = 5,700, 71.12%) had no birth plan documented. Of the 2,315 women who had a birth plan, 1,035 (44.71%) said that it was respected, 601 (25.96%) said that it was not respected and 679 (29.33%) said that they were convinced by the doctor and/or midwife to change their birth plan during their labour. One-third of women (*n* = 2,585, 34.89%) said that the health professional(s) who cared for them in labour introduced themselves when they entered the labour room or when they met them for the first time, and a further 49.61% (*n* = 3,675) said that some of the health professional(s) did this; however, 1,148 of the women (15.50%) said that the health professional(s) looking after them never introduced themselves.

The majority of women were in labour (in hospital) on their first birth for more than 10 h (*n* = 2,858, 38.56%), with a further 1,705 (23%) in labour for over six and up to 10 h and 2,071 women (28.34%) in labour for over two and up to six hours. Most women (*n* = 5,811, 79.78%) felt that they were given privacy in the first stage of labour, but 1,473 women (20.22%) did not. Less than 40% of women had a midwife or other healthcare professional staying with them and supporting them during labour all of the time except for short breaks (*n* = 1,245, 17.36%) or most of the time (*n* = 1,565, 21.82%). The majority (*n* = 3,846, 53.63%) said they only had visits from the midwife to check how they were and 284 (3.96%) said they did not have a midwife caring for them at all.

## Discussion

Data from the 8,836 women who had birthed in one of the 93 hospitals in the Czech Republic, or at home, shed light on women’s recent experiences of prenatal and early labour and birth care in the Czech Republic. Overall, our descriptive statistics show the frequency of aspects of care and interventions performed, and women’s qualitative comments provide rich context on these. The inclusion of the proportions of comments provided serves to show that many women’s experiences are, in some instances, common practices.

We asked women minimal information on the content of their pregnancy care because of the variation in the scheduling and content of care provided in the Czech Republic, but it was clear that abdominal examination was not performed regularly, with 39% of women stating that they never had one performed. It is possible that many of these women had ultrasound scans performed throughout their pregnancy, instead of clinical examination. Forty percent of women were never given information by staff caring for them on where or how they might give birth, a finding in common with a number of studies from other countries [28, 29]. Information-giving on available places of birth has been identified as an essential component of women’s autonomy [30], a key tenet of respectful care. A study exploring satisfaction with maternity care in the Czech Republic, with 1,195 respondents, also found that ‘information giving’ and an ‘empathic and respectful approach’ were lacking, and the aspect rated lowest (34%) was ‘control and involvement in decision-making’ [31].Women who have had a poor previous childbirth experience may be driven away from formal healthcare to give birth alone, if they are not provided with, and are aware of, other possibilities such as homebirth, midwife-led care or birthing centres [32]. Even when women are aware of other options, it may be difficult, if not impossible, to find an alternative healthcare provider or facility because of limited options. It may also be prohibitively expensive for women to employ a private midwife when the fee is not covered by insurance. The lack of discussion, together with unsatisfying conditions in hospitals [31], are the main reasons why women choose homebirth in the Czech Republic [9].

Only one quarter of women in this study were given information on how they might keep their labour normal or non-interventionist as it progressed. Given the documented ill-effects of too much intervention given too soon in labour [11], this is a key area for the provision of information for women, to increase their empowerment and self-efficacy.

The overall induction of labour rate of 25% among this group of women is similar to many other European countries [33]. Induction of labour, when necessary, is a useful and important method of care. However, half of all women who had their labour induced said this occurred before or at term, with a further 25% having labour induced by 41 weeks’ gestation, despite the recommendation from the Czech Gynaecological and Obstetrical Society (CGOS) [34] that there ‘*should be steps taken to end pregnancy in between 41 and 42 weeks*’, so that ‘*pregnancy should be ended by 42 weeks + 0 days’* (not 41 weeks + 0 days). Interpretation of this recommendation by individual healthcare providers is permitted, and earlier definitions of ‘term’ appear to be used frequently.

Qualitative comments from the women also seemed to indicate confusion around the meaning of ‘term’ and ‘post-term’. It is questionable that 75% of all the women who had induction of labour before the ‘post-term’ period, as recommended by the CGOS, required it for a genuine medical, obstetrical or fetal reason. Considerable variation is seen across the world in induction of labour rates, with no difference in outcomes, indicating that some inductions are unnecessary [35], as seems to be the situation in the Czech Republic. Induction of labour for subjective, non-medical, reasons was also noted in research from the United States [36], and is linked in a number of countries with increasing CS rates [35].

Just over half of the women who had labour induced thought that the reason that this was necessary was discussed sufficiently with them, but 55% had not been given any choice in the decision. The greater involvement of obstetricians at all levels of care in the Czech Republic may account for this lack of choice, as other countries such as the US [37] have shown that midwifery care encourages women’s decision-making.

When labour is induced by artificial rupture of membranes and/or use of oxytocin infusion or prostaglandin pessaries, the woman is aware of the procedure in advance and, by presenting at the hospital on the date requested, has given tacit (and, usually, written) consent. Thirty-five percent of women in this study had labour induced by the Hamilton manoeuvre (sweeping the membranes) which can be effective in achieving a spontaneous onset of labour and can potentially reduce the incidence of a more formal method of induction of labour [38]. However, 56% of the 1,364 women who had the Hamilton manoeuvre performed said that the doctor had not asked for their permission beforehand. This is an example of very disrespectful care and may be occurring, unacknowledged, in many other countries as a recent systematic review [39] failed to find any research in this area. Not gaining women’s consent for interventions or procedures is not uncommon, as is shown in systematic reviews [40, 41] and in studies conducted in a myriad of settings globally [15–17, 41–43].

The majority of women had a companion of their choice with them throughout labour. However, 11% of women were only permitted to have their companion with them for a short time at the start, or end, of labour, a practice that should never occur; having a companion to support one in labour is a basic human right, upheld by the WHO [44]. An obstetrician/gynaecologist or doctor provided intrapartum care for 77% of women, and 75% said that they were cared for by a midwife instead of, or as well as, an obstetrician. However, the majority of women (54%) said that they ‘*only had visits*’ from the midwife to check how they were, with no-one present with them throughout the birth process, and 4% said that they did not have a midwife caring for them at all. Lappeman and Swartz’s [12] qualitative study, which used labour ward observations as the primary data collection method, revealed the ‘silence of the labour ward’ and the ‘neglect of the neglect’ where women in labour ‘lay in beds alone’, rarely with companions. Research findings from across the world show that women who receive continuous labour support (from midwives, doulas, or lay companions), especially from one or two known midwives, are more likely to have shorter labours with less use of pain medication, less intervention, birth spontaneously [37, 45, 46]and be more satisfied [47–49].

Other instances of non-respectful care are revealed in the responses from 16% of women who said that clinicians looking after them never introduced themselves when they entered the labour room or met them for the first time. Analysis of the psychosocial climate in maternity hospitals in the Czech Republic similarly indicates the need for enhanced communication skills by healthcare providers, especially when communicating consistent information [31, 50]. Less than one-third of women had a birth plan but over half of those who had said their plan was not respected or that they were persuaded by the clinician to change their plan during labour. In addition, 20% were not given privacy in the first stage of labour, similar to findings from other studies in Jordan [51] and Turkey [52]. Experiencing privacy and healthcare provider courtesy in labour have been shown to be key determinants of maternal satisfaction, in a review of 54 research papers from low-income countries [53].

Overall, our quantitative and qualitative data reveal aspects of disrespectful prenatal and intrapartum care. Taken in their entirety, there was a considerable number of women who had no information on and no choice in their place of birth, procedures and interventions performed without explanation or consent and who were ‘persuaded’ to alter their plan for their care. Our qualitative comments very much resonate with those in Kuipers et al.’s [18] study which explored the experiences of women, including women who birthed in the Czech Republic, who had a negative or traumatic birth and the value, sense and meaning assigned to the social space of birth. The authors stated that women frequently experienced their birth environment as coercive and disrespectful, described being ‘physically forced into positions or spaces’ repeatedly (p4) and an environment that depicted ‘scenes of horror’ (p5).

Cohen Shabot [54] characterises obstetric violence, and any and all forms of disrespecting pregnant women, as a feminist issue which must be examined through feminist views on violence i.e., violence directed at women and part of a general patriarchal oppression of women. She describes it as gender violence because women are its main victims. According to Šmídová et al. (2015), the privately-held and shared convictions amongst obstetricians that change is needed in healthcare relating to childbirth contrasts markedly with their reluctance to say this publicly and be critical of the system. Whilst the opinions of the obstetricians, those who dominate and currently hold the power within the maternity hospitals and services, remain polarised and open discussions are absent, implementation of a woman-centred approach to care and service may be slow. On a more positive note, the Government’s Gender Equality Strategy for 2021–2030 acknowledges that there has long been a strong social demand for a respectful approach and humanisation of obstetrics care for women (acknowledged as being most women) who experience physiological pregnancy and birth (most women). It also states that the Ministry of Health has begun to respond to this by working on the concept for supporting the establishment of midwifery centres within maternity hospitals [55]. The Strategy also recognises the persistent legislative and restrictions that midwives have long faced and the resulting negative impact on women and their families in terms of choice of place, method and circumstances of childbirth. It also acknowledges that this is further complicated by the absence of national standards of care, the links between the various professions that offer peripartum care, and the repeated criticisms from international institutions and others.

### Strengths and limitations

A strength of this study is that data were provided by a large sample: 8,836 women who had experienced birth in one of the 93 hospitals in the Czech Republic, or at home. The inclusion of women’s qualitative comments, including proportions when relevant, provide rich and detailed context on interventions and procedures. The main limitation is that surveys were completed by a self-selecting sample of women, or their partners, and are therefore potentially biased. Whilst we sought to recruit participants from a wide range of sources, including maternity hospitals, obstetric and gynaecology clinics, postnatal centres, kindergartens etc., it is possible that the experiences reported here may not be representative of women birthing in the Czech Republic. Whilst 51% of respondents had birthed their baby more than one year previously, which may raise issues of recall bias, the accuracy of women’s memories of their experiences, and their agreement with data recorded in their maternity care records [56] even up to five years after the birth, has been documented [57]. Whilst the views of 69 partners were included, partners answered the questions in relation to their partner’s experiences. We did not analyse these separately but including their information about their partners was deemed important.

## Conclusions

Findings reveal a lack of information-giving, discussion and shared decision-making from healthcare professionals in the maternity care services. There are also some indications that some practices were not based on evidence. A thorough audit of clinical practices, in individual hospitals and nationally, and the open publication on labour and birth outcomes, and women’s experiences of care, is needed. Women described unacceptable aspects of care such as having procedures performed without their consent, and concerns around discourtesy of healthcare professionals and lack of privacy. Without doubt, these experiences cause women great distress during, and even after, childbirth. Our findings highlight areas in need of urgent improvement in maternity care services in the Czech Republic. Initiatives such as the Ministry of Health‘s 2015 call for comments from parents on their experiences of services are to be commended and continued, as is the implementation of the Government’s Gender Equality Strategy and other initiatives that target women-centred reformations within the maternity care system.

### Electronic supplementary material

Below is the link to the electronic supplementary material.

Supplementary Material 1

Supplementary Material 2

## Data Availability

All data generated or analysed during this study are included in this published article [and its supplementary information files].
