# Supplementary material for: An online survey of women’s views of respectful and disrespectful pregnancy and early labour care in the Czech Republic
Source: BMC Pregnancy Childbirth. 2024 May 15;24:370. doi: 10.1186/s12884-024-06448-5 (PMC11097455; doi:10.1186/s12884-024-06448-5)
Supplement: Supplementary file 1 — Supplementary Material 1 [file 12884_2024_6448_MOESM1_ESM.docx]

**S1** **Table Number and proportion of respondents from each hospital**

| **Percentage of total responses** | **Hospital, number of respondents and percentage of total responses** |
| --- | --- |
| 3.00-7.00% | ÚPMD Podolí (n=528, 6.02%); Porodnice Praha Apolinář (n=411, 4.68%); Porodnice Plzeň - Fakultní nemocnice (n=317, 3.61%) |
| 2.00-2.99% | Motol (n=260, 2.96%); Bohunice (n=252, 2.87%); Porodnice Brno - Obilný trh (n=241, 2.75%); Milosrdných bratří (n=213, 2.43%); Porodnice České Budějovice (n=179, 2.04%); Porodnice Hořovice (n=179, 2.04%) |
| 1.00-1.99% | Fakultní nemocnice (n=174, 1.98%); Porodnice Hradec Králové (n=173, 1.97%); Porodnice Zlín (n=172, 1.96%); Na Bulovce (n=168, 1.91%); Krč,Thomayerova nemocnice (n=159, 1.81%); Vítkovická nemocnice (n=158, 1.80%); Porodnice Olomouc (n=149, 1.70%); Porodnice Jablonev nad Nisou (n=146, 1.66%); Porodnice Neratovice (n=146, 1.66%); Porodnice Ústí nad Labem (n=134, 1.53%); Porodnice Pardubice (n=129, 1.47%); Porodnice Ostrava- Městská nemocnice (n=129, 1.47%); Liberec (n=125, 1.42%); Porodnice Vyškov (n=121, 1.38%); Porodnice Mělník (n=119, 1.36%); %); Porodnice Rakovník (n=117, 1.33%); Porodnice Havlíčkův Brod (n=112, 1.28%); Porodnice Chrudim (n=100, 1.14%); Porodnice Uherské Hradiště (n=93, 1.06%); Porodnice Mladá Boleslav (n=91, 1.04%); Porodnice Krnov (n=91, 1.04%); Porodnice Ústí nad Orlicí (n=89, 1.01%) |
| 0.06-0.99% | Porodnice Kolín (n=87, 0.99%); Královské Vinohrady (n=87, 0.99%); Porodnice Příbram (n=86, 0.98%); Porodnice Nymburk (n=84, 0.96%); Porodnice Tábor (n=82, 0.93%); Porodnice Kladno (n=82, 0.93%); Porodnice Jilemnice (n=78, 0.89%); Porodnice Kyjov (n=75, 0.85%); Porodnice Třebíč (n=75, 0.85%); Porodnice Jičín (n=74, 0.84%); Porodnice Šternberk (n=72, 0.82%); Porodnice Jihlava (n=71, 0.81%); Porodnice Písek (n=65, 0.74%); Porodnice Český Krumlov (n=63, 0.72%); Porodnice Kadaň (n=63, 0.72%); Porodnice Most (n=63, 0.72%); Porodnice Břeclav (n=62, 0.71%); Porodnice Opava (n=62, 0.71%); Porodnice Ivančice (n=62, 0.71%); Porodnice Strakonice (n=61, 0.70%); Porodnice Karviná (n=61, 0.70%); Porodnice Frýdek Místek (n=60, 0.68%); Porodnice Litoměřice (n=60, 0.68%); Porodnice Teplice (n=55, 0.63%); Porodnice Klatovy (n=54, 0.62%); Porodnice Nové město na Moravě (n=54, 0.62%); Porodnice Šumperk (n=54, 0.62%); Porodnice Ostrov (n=54, 0.62%); Porodnice Vsetín (n=52, 0.59%); Porodnice Náchod (n=51, 0.58%); Porodnice Rychnov nad Kněžnou (n=51, 0.58%); Porodnice Kroměříž (n=50, 0.57%); Mulačova nemocnice (n=50, 0.57%); Porodnice Česká Lípa (n=49, 0.56%); Porodnice Znojmo (n=47, 0.54%); Porodnice Pelhřimov (n=46, 0.52%); Porodnice Valašské Meziříčí (n=45, 0.51%); Porodnice Přerov (n=44, 0.50%); Porodnice Slaný (n=44, 0.50%); Porodnice Jindřichův Hradec (n=43, 0.49%); Porodnice Boskovice (n=43, 0.49%); Porodnice Karlovy Vary (n=42, 0.48%); Porodnice Nový Jičín (n=41, 0.47%); Porodnice Stod (n=41, 0.47%); Porodnice Roudnice nad Labem (n=40, 0.46%); Porodnice Svitavy (n=40, 0.46%); Porodnice Třinec (n=40, 0.46%); Porodnice Chomutov (n=38, 0.43%); Porodnice Trutnov (n=37, 0.42%); Porodnice Sokolov (n=35, 0.40%); Porodnice Cheb (n=33, 0.38%); Porodnice Děčín (n=32, 0.36%); Porodnice Prostějov (n=32, 0.36%); Porodnice Rokycany (n=31, 0.35%); Porodnice Čáslav (n=26, 0.30%); Porodnice Domažlice (n=25, 0.28%); Porodnice Prachatice (n=23, 0.26%); Porodnice Vrchlabí (n=21, 0.24%); Porodnice Rumburk (n=12, 0.14%); Porodnice Brandýs nad Labem (n=12, 0.14%); Porodnice Rudolfa a Stefanie Benešov (n=7, 0.08%); Porodnice Turnov (n=5, 0.06%) |
