# Supplementary material for: An online survey of women’s views of respectful and disrespectful pregnancy and early labour care in the Czech Republic
Source: BMC Pregnancy Childbirth. 2024 May 15;24:370. doi: 10.1186/s12884-024-06448-5 (PMC11097455; doi:10.1186/s12884-024-06448-5)
Supplement: Supplementary file 2 — Supplementary Material 2 [file 12884_2024_6448_MOESM2_ESM.doc]

**S2: Survey on Respectful Care in Czech Republic** (Survey administered online)

For mothers, fathers and other interested persons

Thank you for participating in our survey. Your feedback is important, and will be used to improve maternity care in the Czech Republic.

We are conducting an exploratory study of the level of respectful or non-respectful care given to women during pregnancy and birth in the Czech Republic. We would like to hear your views on this. Researchers [Authors). If you have any queries about the study please feel free to contact [Author’s email address].

Any information you provide in this survey is anonymous and we encourage you to answer the questions honestly. Participation in this survey is entirely voluntary. **We would like you to answer this survey, based on your experiences of your FIRST birth;** however, if you have had more than one baby, you are very welcome to make comments after each question, relating to another birth (or all your births).

If you are working in maternity care and are NOT a woman who has had a baby in the Czech Republic, a father of a baby born in the Czech Republic or another person interested in maternity care, please do NOT continue to fill in this questionnaire.

If you are working in maternity care AND are also either a woman who has had a baby in the Czech Republic, or a father of a baby born in the Czech Republic, please DO continue to fill in this questionnaire with your experiences of your own/your partner’s birth.

Ethical principles that apply to research will be adhered to throughout the study, and ethical approval has been obtained from [Name of Research Ethics Committee].

Please note that completion of the survey is taken as consent to participate in the study. If you are able to answer in English, when making comments, please do so.

1) I have read and understood the information provided

Yes

No

1. Are you

A woman who has had a baby in the Czech Republic

A father of a baby born in the Czech Republic (please answer the questions in relation to your partner’s experience, and provide comments on your own experience in the comment boxes)

Another person interested in maternity care (please specify below)

A midwife registered in the Czech Republic

A doula working in the Czech Republic

Other healthcare worker providing maternity care in the Czech Republic (please specify below)

1. What is your age range? (If you are 17 or younger, please do not continue to complete the survey).


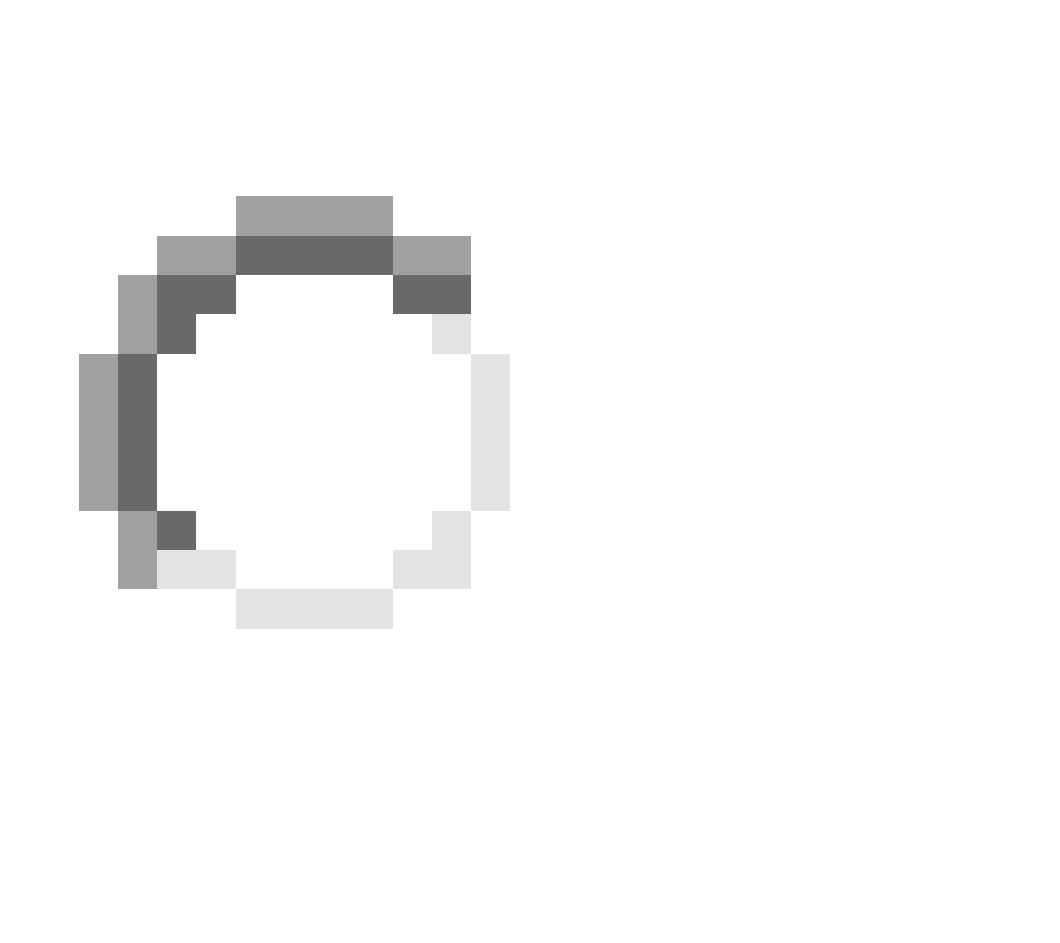
17 or younger


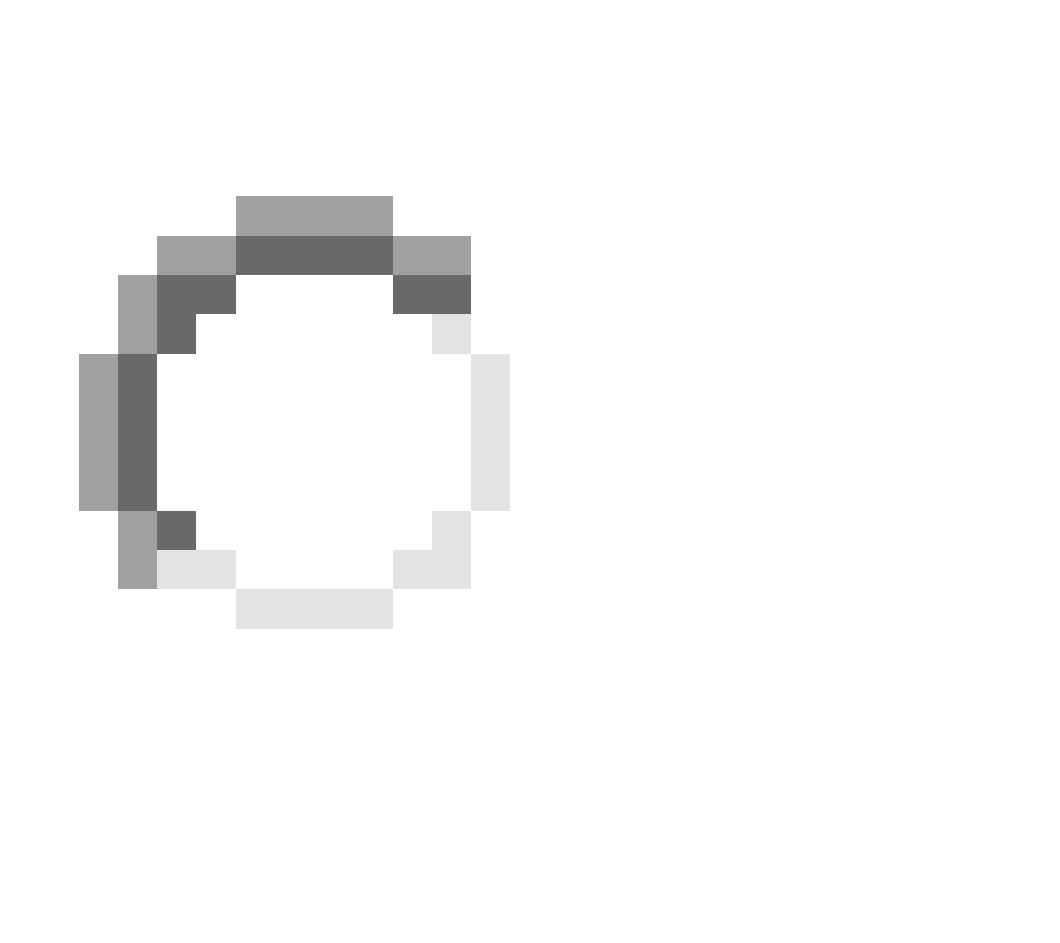
18-25


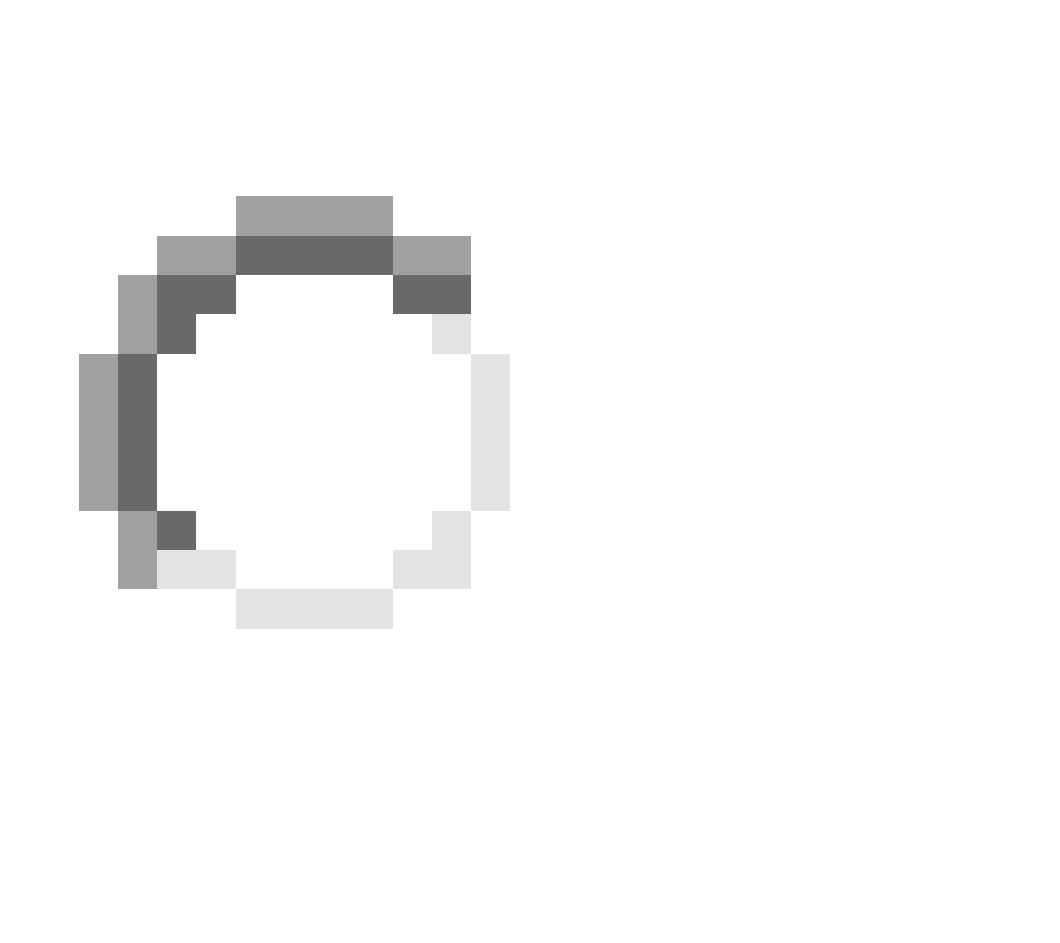
26-30


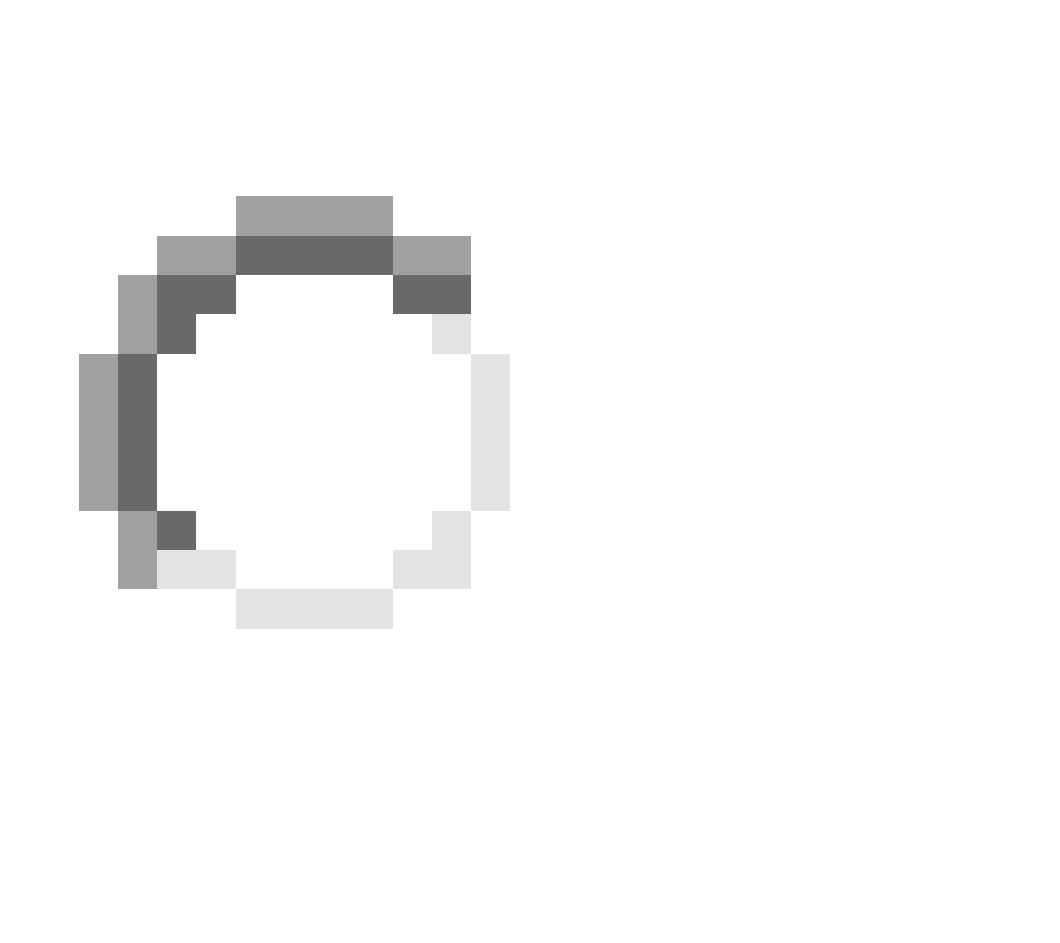
31-35


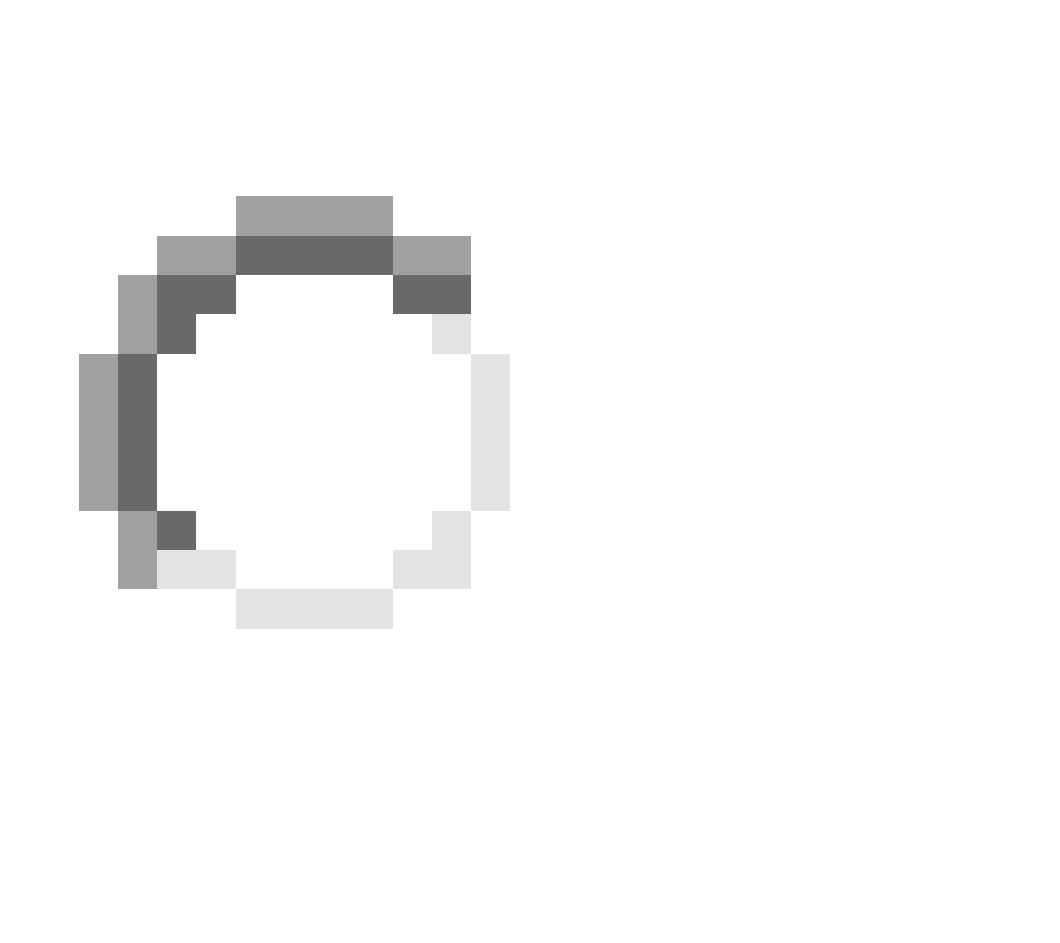
36-40


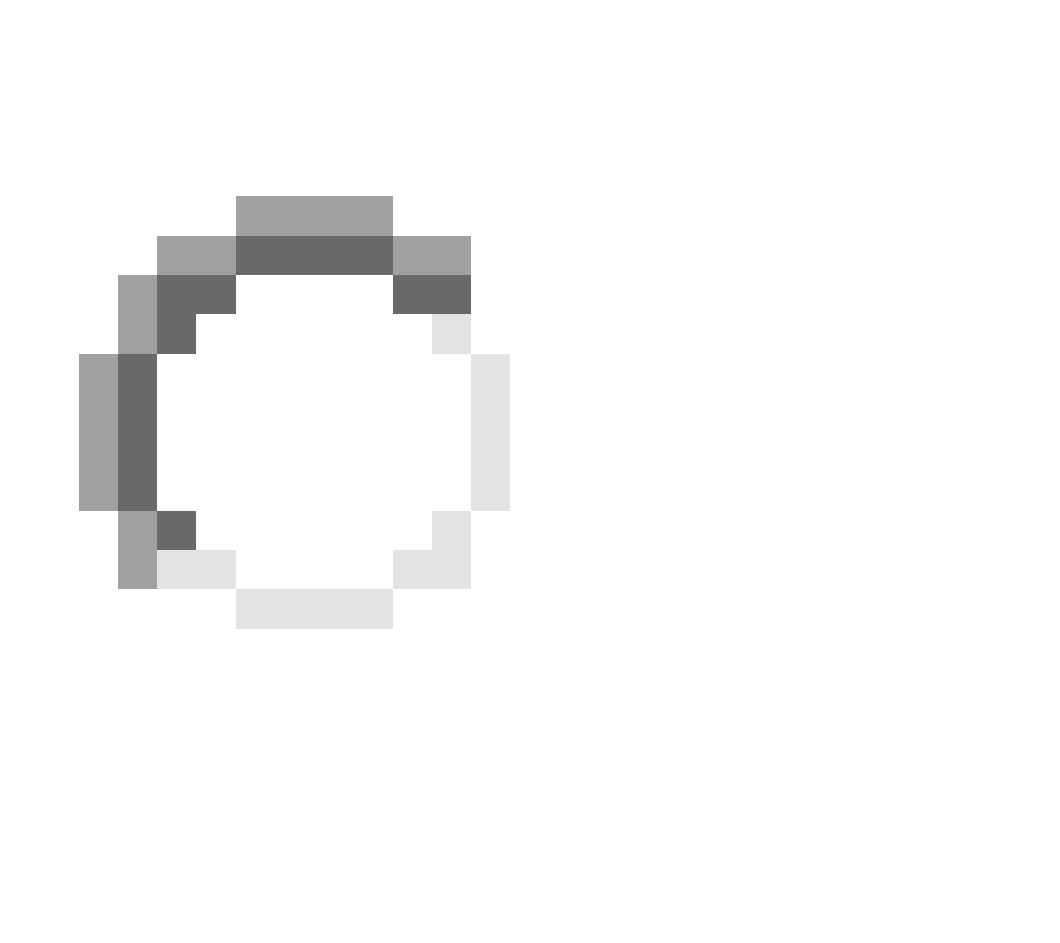
40 or older

4. How old is your youngest baby?


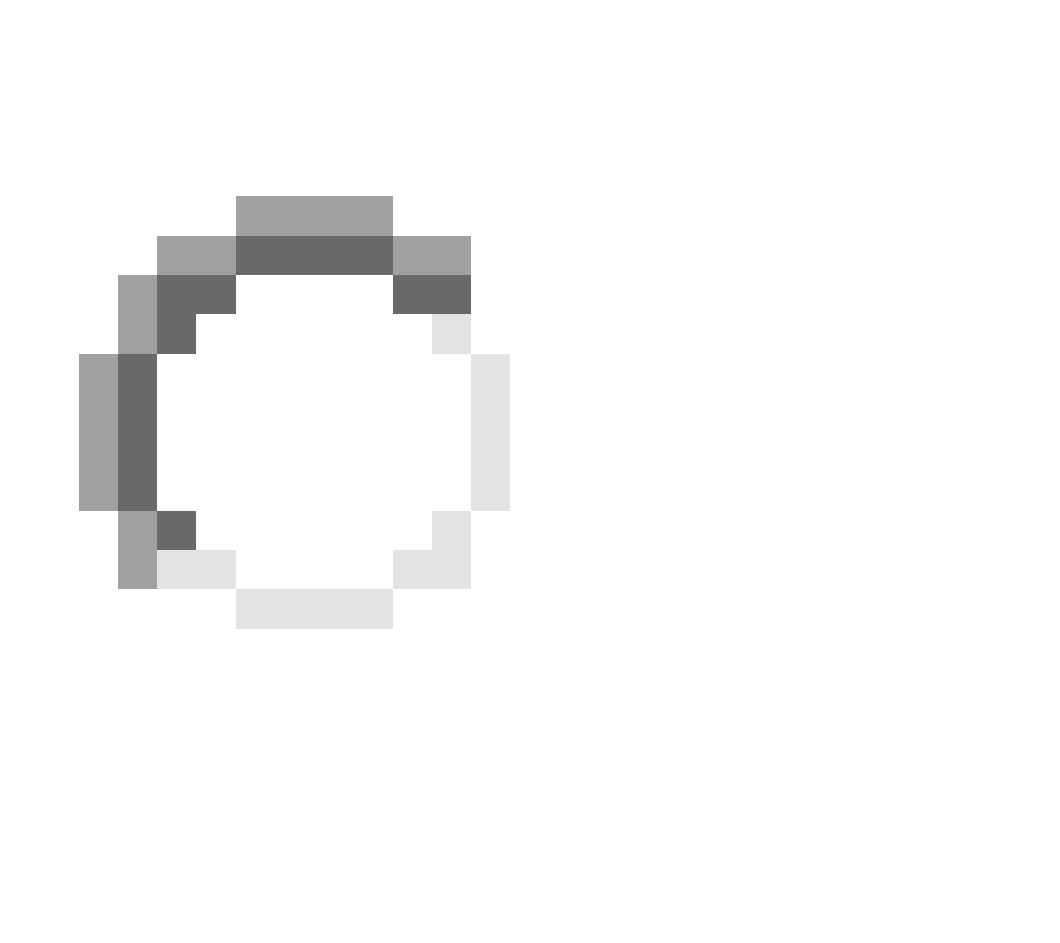
Less than 1 month old


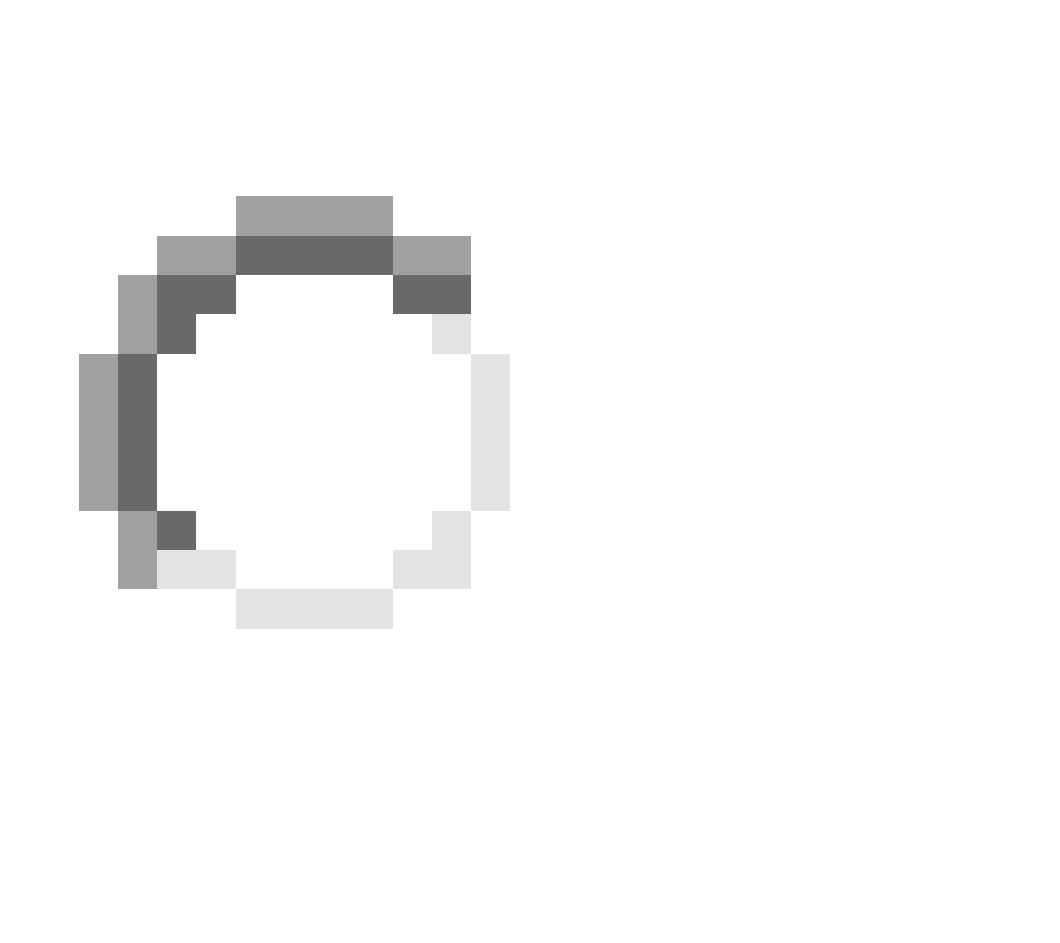
1 month – less than 6 months old


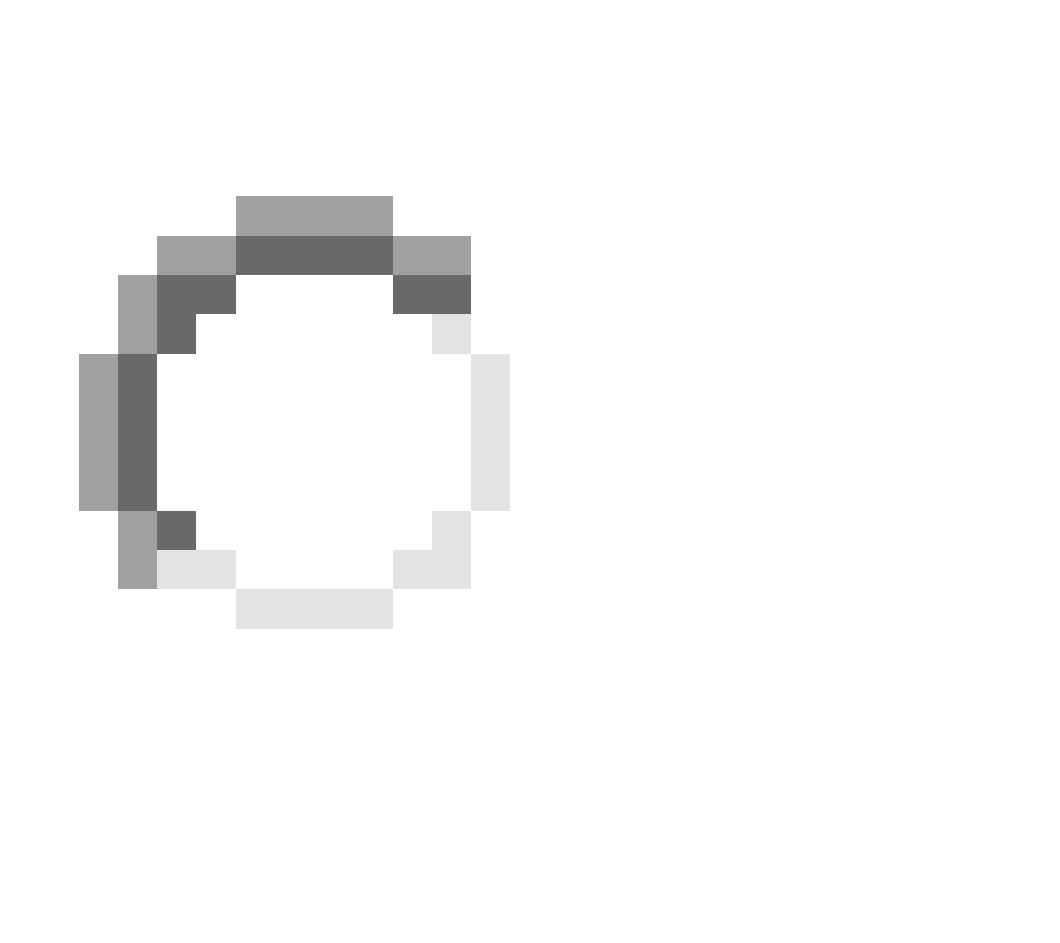
6 months – less than one year


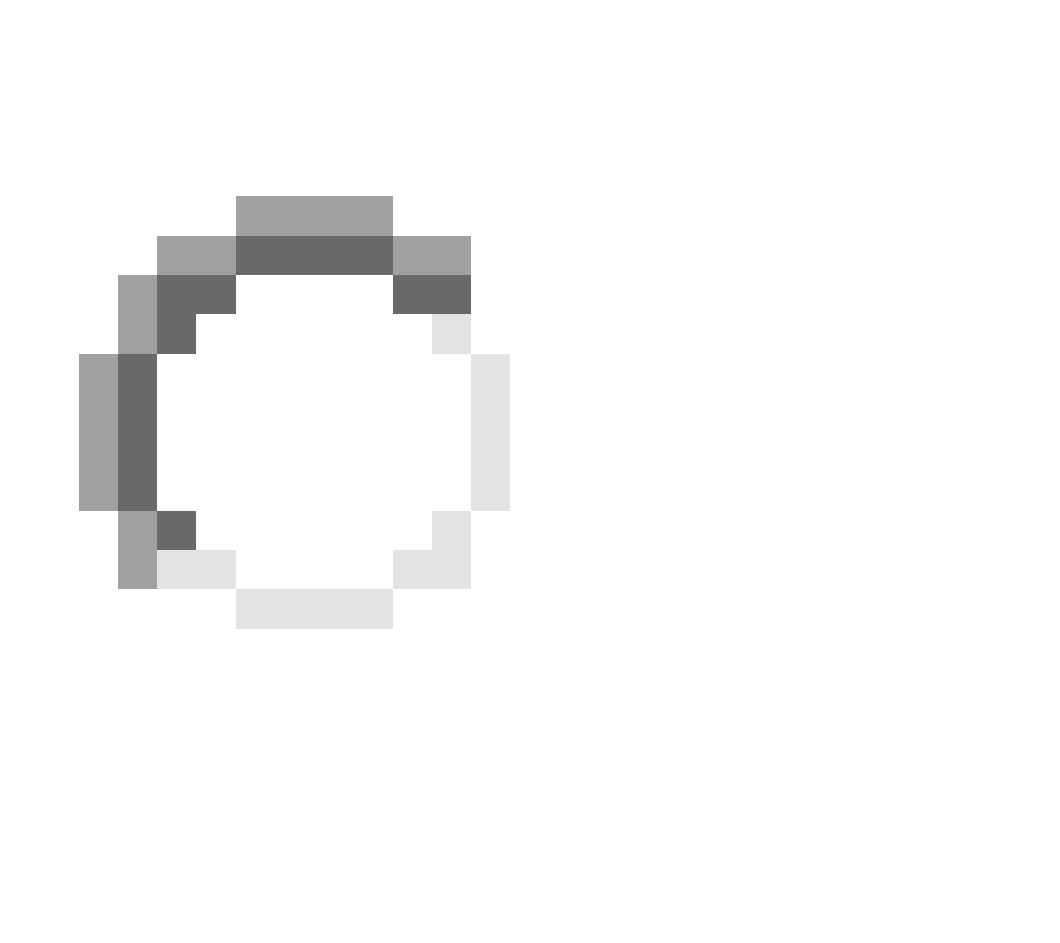
Older than one year


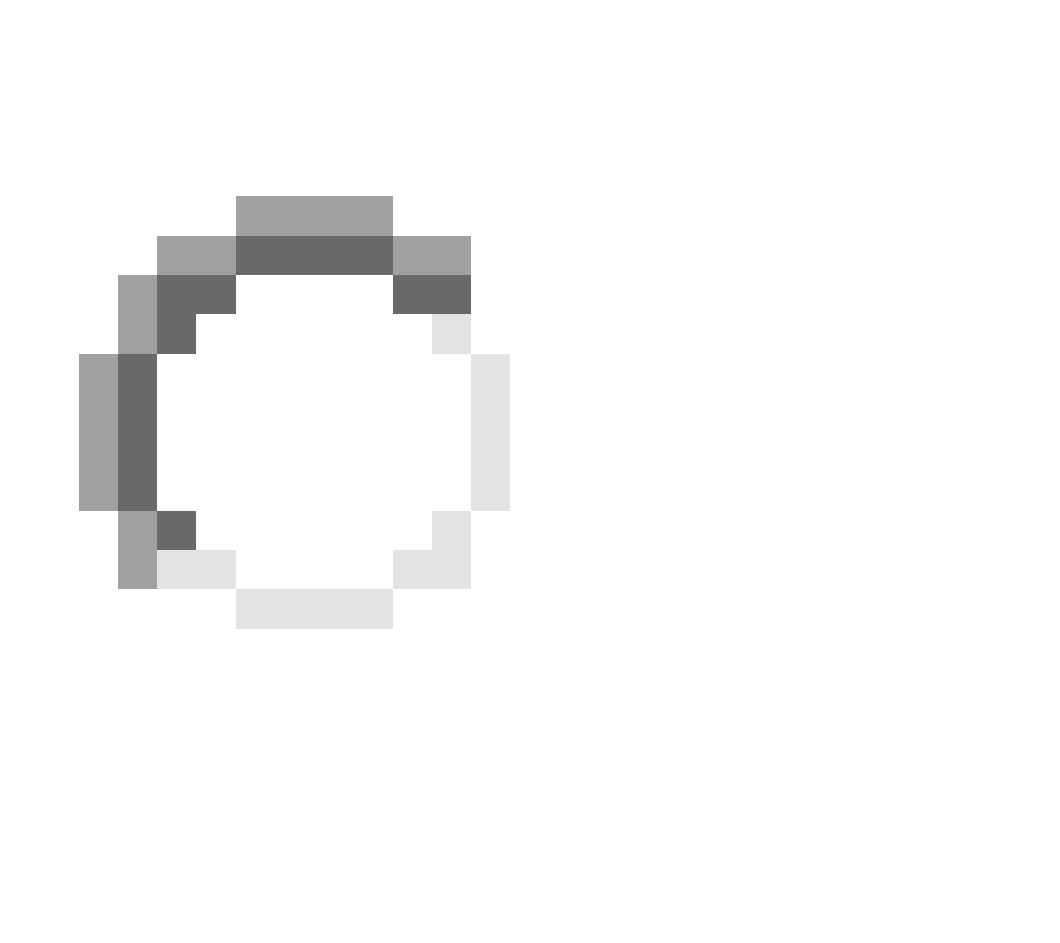
Have not had a baby (please ignore all questions on birth but provide comments in comment boxes in relation to the issues raised)

5. How old is your oldest child?


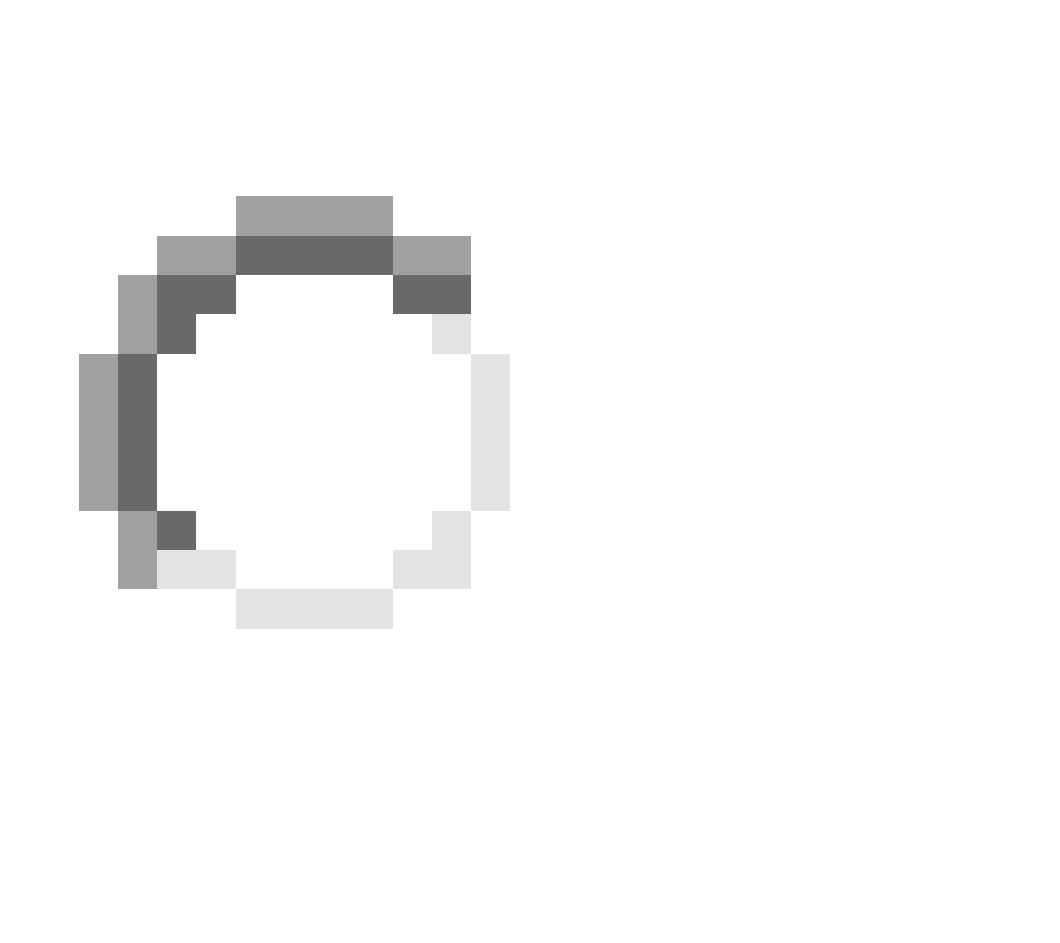
Have no other children


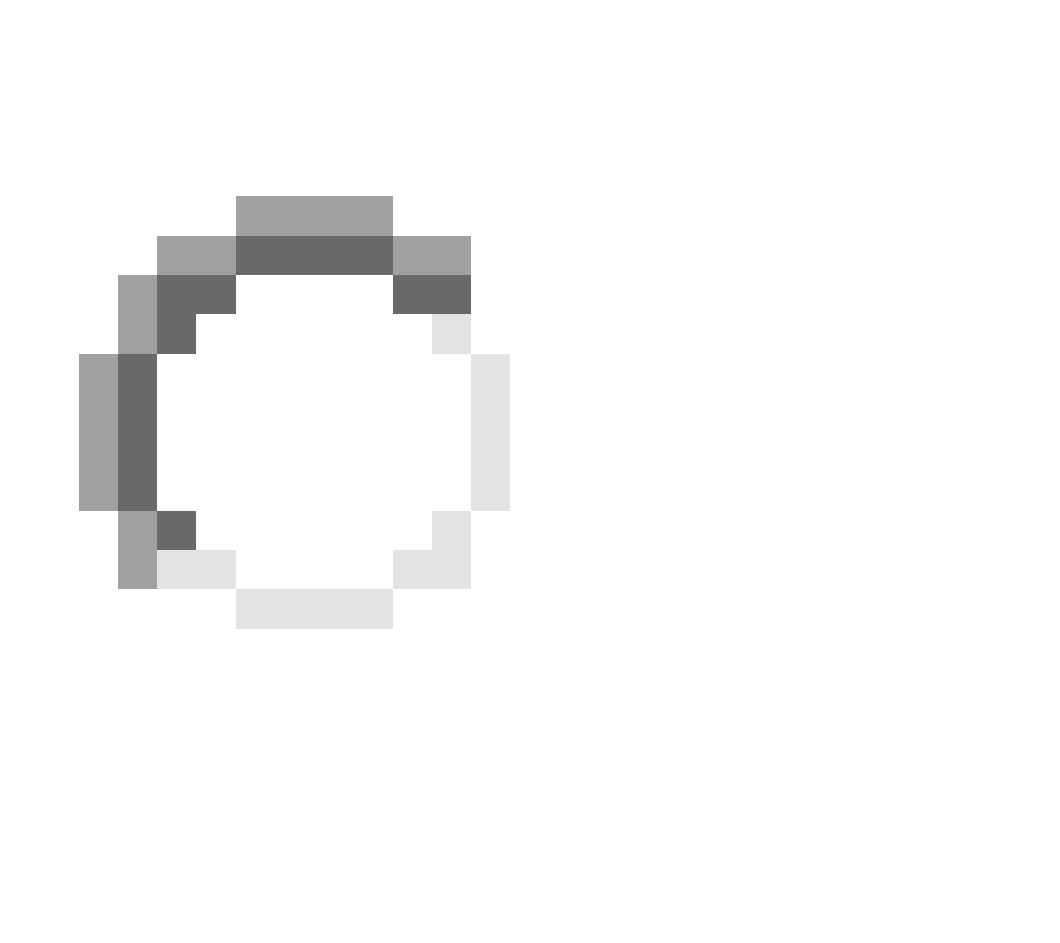
1 - 2 years old


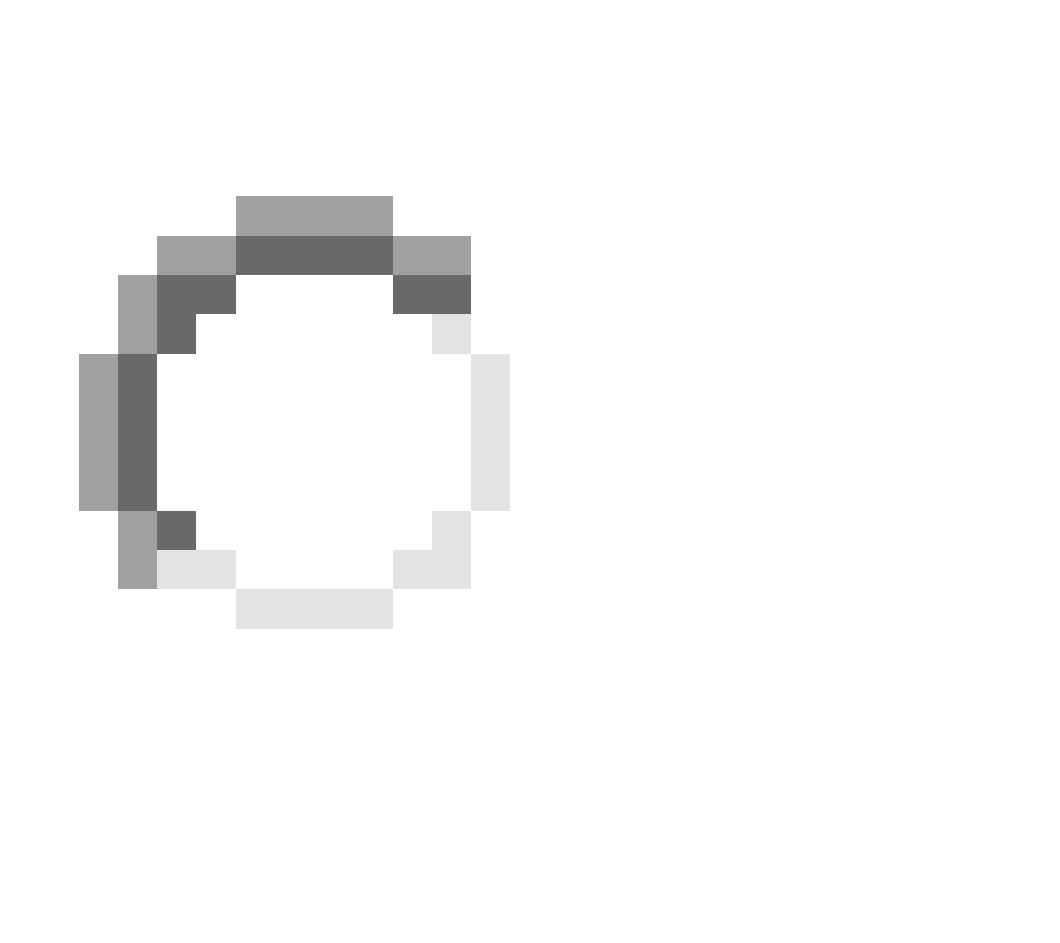
3 - 4 years old


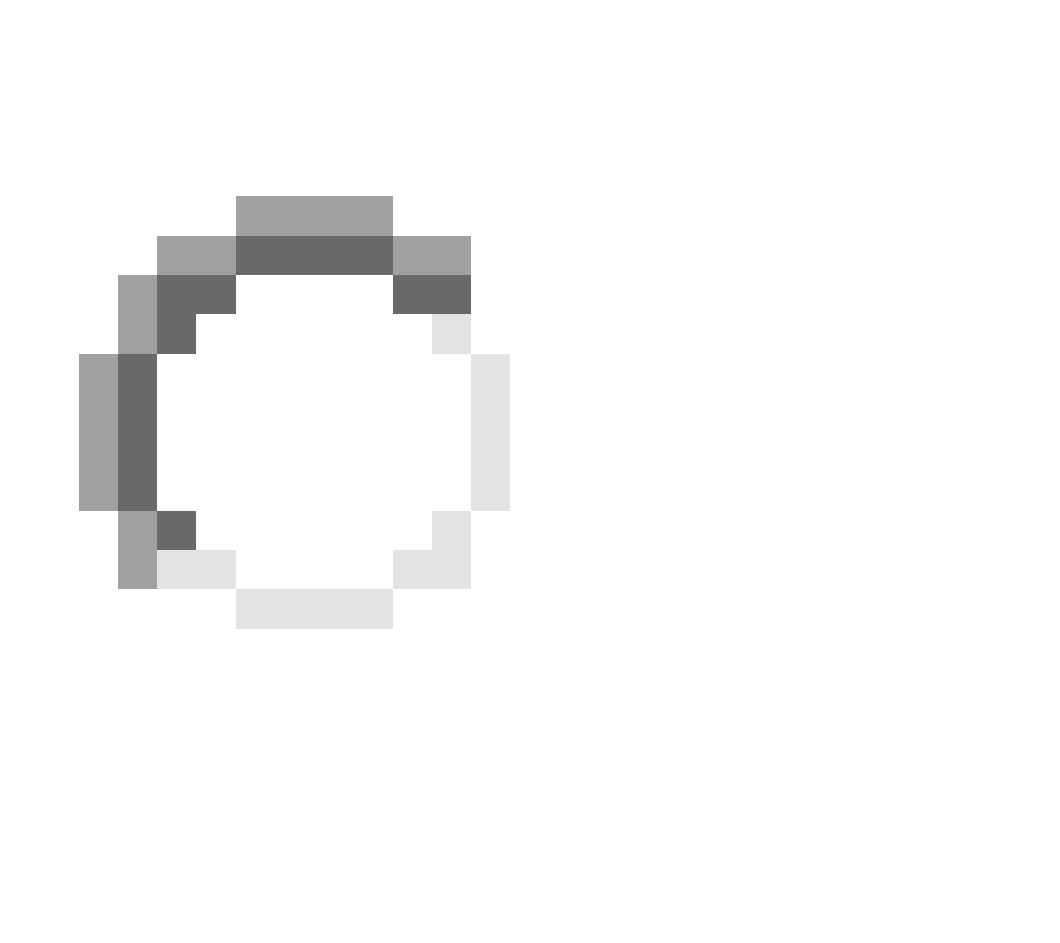
5 – 6 years old


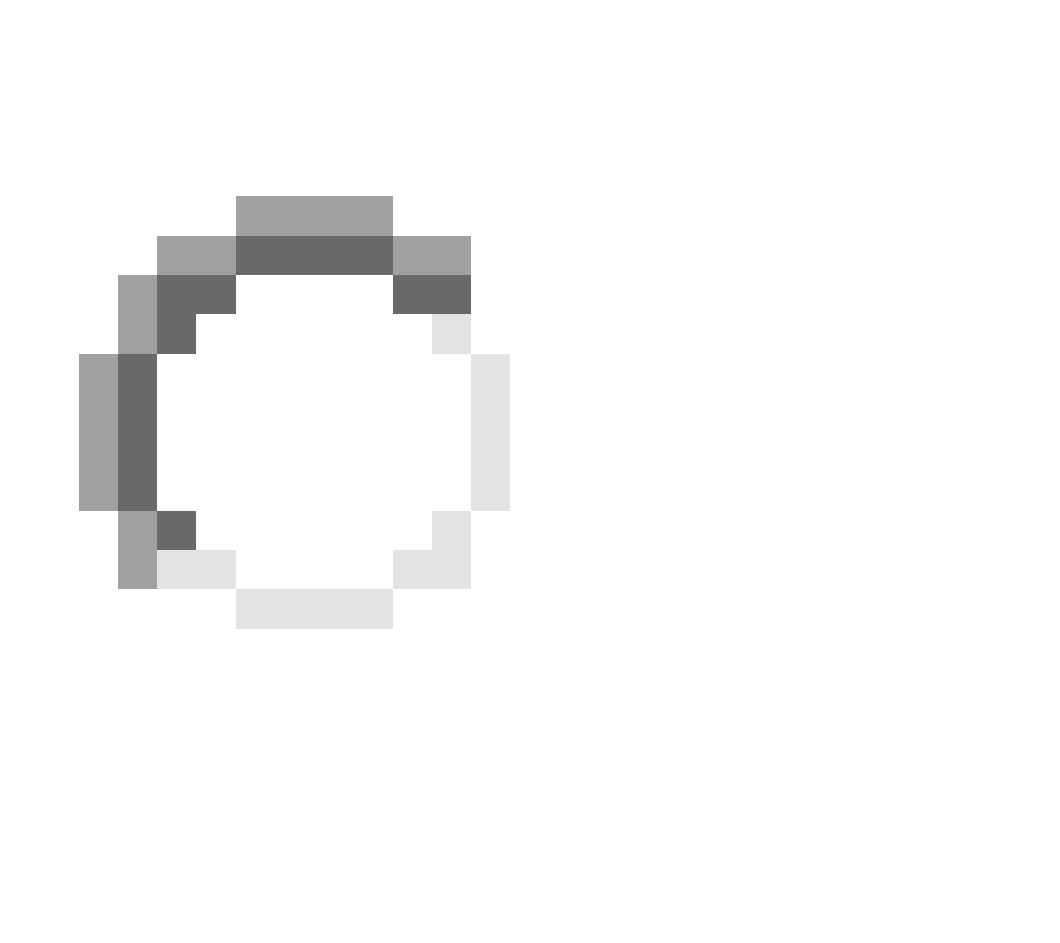
7 - 8 years old


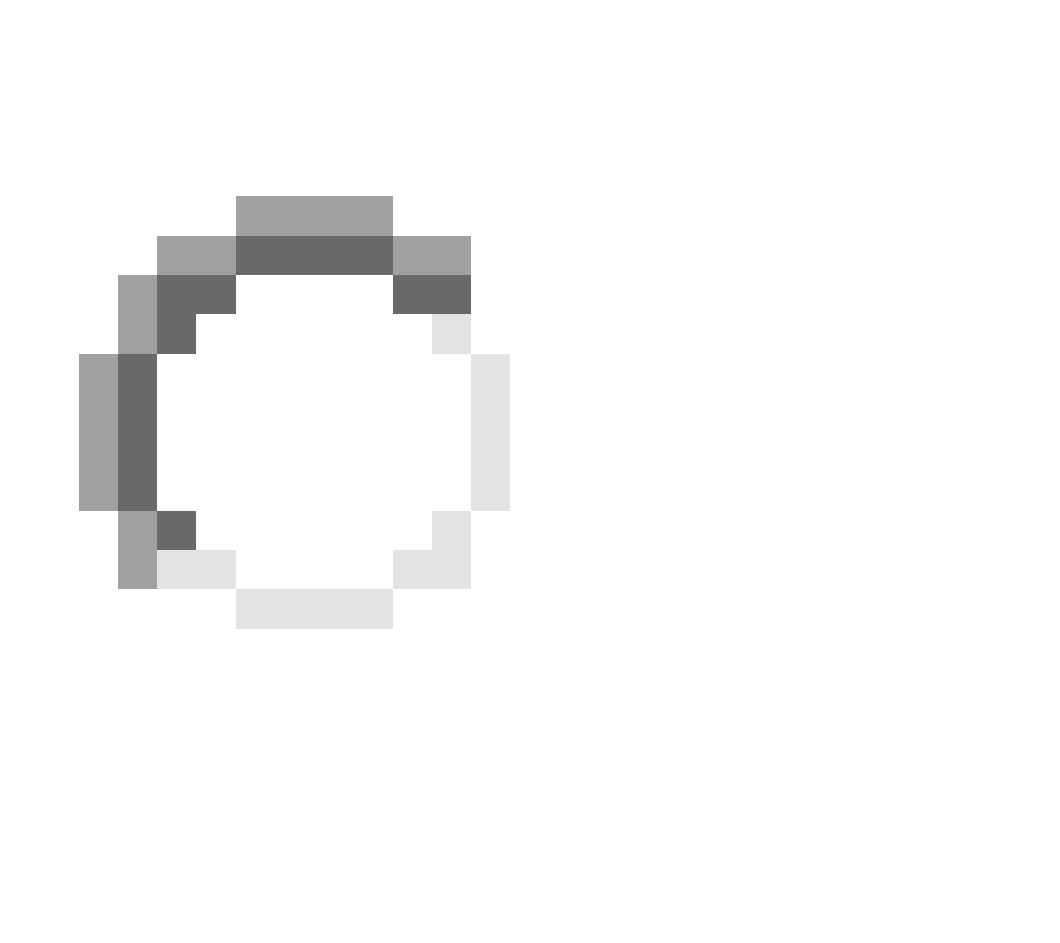
Older than 8 years

6. In which hospital did you give birth to your first baby? (please tick Home if you had a home birth).

Hospital A

Hospital B…..

Home

Comments

7. Who took care of you during your first labour?

-obstetrician/gynaecologist/doctor

-obstetrician/gynaecologist/doctor, with extra payment given directly to them (please state how much in the text box)

-hospital midwife

- private midwife, allowed to care for you throughout labour in hospital

- private midwife, allowed to care for you in hospital, but working as a doula only

- private midwife who does not have a contract with hospital

-doula

- other (please explain in the comment box)

-unsure

- no-one

Comments

8. During your first pregnancy did your obstetrician/gynaecologist, the staff in the hospital, or your (home birth) midwife or doula, perform an abdominal examination (touch your tummy to see how the baby was growing)

Yes, once or twice

Yes, on every visit

No, never

Comments

9. During your first pregnancy did your obstetrician, the staff in the hospital, or your (home birth) midwife or doula, give you information about

|  | **Yes** | **No** | **Can't remember** |
| --- | --- | --- | --- |

Choices available for place of birth

Choices available for mode of birth

Positions you could use for labour and birth

The benefit and importance of labour remaining natural or noninterventionist

How to keep labour remaining natural or noninterventionist

Comments

[Reminder: all the following questions are about your FIRST birth. If you would like to make comments about any subsequent births, please make them in the comment box]

10. Did your labour start naturally (at or after your due date)?

Yes No

 Comments

| 11. If No, what was the reason given to you for not waiting for labour to start naturally?  Comments  12. If No, did you have induction of labour (labour started artificially), or an elective (planned) caesarean section?  induction of labour                     elective caesarean section  13. If you had your labour induced, when was this done?  Not applicable (labour not induced)  Before term  At term (exactly 40 weeks)  40 weeks plus 1 or 2 days  40 weeks plus 3 or 4 days  40 weeks plus 5 or 6 days  41 weeks  41 weeks plus 1 or 2 days  40 weeks plus 3 or 4 days  40 weeks plus 5 or 6 days  At exactly 42 weeks  Over 42 weeks  Comments      14. If you had an elective (planned) caesarean section, when was this done?    Not applicable (did not have CS)  Before term  At term (exactly 40 weeks)  40 weeks plus 1 or 2 days  40 weeks plus 3 or 4 days  40 weeks plus 5 or 6 days  41 weeks  41 weeks plus 1 or 2 days  40 weeks plus 3 or 4 days  40 weeks plus 5 or 6 days  At exactly 42 weeks |  |
| --- | --- |

15. If you had an induced labour or an elective (planned) caesarean section, was the reason that this was necessary discussed sufficiently with you (all positives and negatives)?

Yes

No

Text box for comments

16. If you had an induced labour or an elective caesarean section, did you feel you were given any choice in the decision, and were you given time to make a decision you were comfortable with?

Yes

No

Text box for comments

17. Did you have a “birth plan” and was it respected?

I had a “birth plan” and it was respected

I had a “birth plan” but it was NOT respected

I was convinced by the doctor and/or midwife to change my “birth plan” while giving birth

I did not have a “birth plan”

Textbox for comments

**(If you had an elective caesarean section, please go now to question 58)**

18. If you had your labour induced, was this done by sweeping the membranes during a vaginal examination but not rupturing membranes (Hamilton manoeuvre), or by rupturing membranes (breaking the waters), or by giving you a drug vaginally (some gel/pastille into your vagina), or by giving you a drug intravenously (by a drip)? Please mark all that apply.

Sweeping the membranes during a vaginal examination (Hamilton manoeuvre)

Rupturing membranes (breaking the waters)

A drug vaginally (some gel/pastille into your vagina)

A drug intravenously (by a drip)

Text box for comments

19. If your labour was induced by sweeping membranes (Hamilton manoeuver) did the doctor ask for your permission before doing this manoeuver?

Yes

No

Not applicable

Text box for comments

20. Did you have a birth companion?

Yes, baby´s father

Yes, doula

Yes, private midwife

Yes, another person (please state below)

No, I didn´t have a companion

Comments

21. Was your birth companion allowed to stay with you for the whole of your labour?

Yes (or yes, apart from some short breaks)

Yes, but only for about half the labour

No, only for a short time at the start, or at the end, of labour

No, not at all

Comments: (You can describe how it was if you like)

22 . Did the health professional(s) who cared for you during your labour, introduce themselves when they entered your labour room or when you met them for the first time?

Always

Some of them did

Never

Comments: (You can describe how it was if you like)

23. How long was your labour and birth (only the part you were in hospital)?

Up to 2 hours

Over 2 hours up to 4 hours

Over 4 hours up to 6 hours

Over 6 hours up to 8 hours

Over 8 hours up to 10 hours

Over 10 hours

24. Did you have privacy during first stage of labour?

Yes     No

Comments: (You can describe how it was if you like)

25. At any stage in labour, was an electronic fetal monitor applied (belts on your tummy), which gave a continuous tracing of the baby’s heart beat?

Yes

No

26. If an electronic fetal monitor was applied during labour, which gave a continuous tracing of the baby’s heart beat, was the reason that this was necessary discussed with you?

Yes

No

Text box for comments

27. If an electronic fetal monitor was applied during labour which gave a continuous tracing of the baby’s heart-beat, were you given any choice in the decision to apply the monitor?

Yes

No

Text box for comments

28. Did you move around freely during labour, or were you lying in a bed for most of your labour?

Moved around freely

Lay in bed

Other (please specify)

29. If you lay in bed for your labour, was this your choice, or was it suggested by healthcare workers, or were you told by healthcare workers that you had to lie in bed?

It was my choice

It was suggested by healthcare workers that **it was best** to lie in bed

I was told by healthcare workers that **I must** lie in bed

Nothing much was explained or recommended to me

Not applicable

Text box for comments

[Reminder: all these questions are about your FIRST birth. If you would like to make comments about any subsequent births, please make them in the comment box]

30. Did you have a midwife or other healthcare professional staying with you and supporting you during your labour?

Yes, all the time except for short breaks

Yes, most of the time

No, only visits to check how I was

No, not at all

Other (please specify)

Comments

31. When you were in labour (at any stage), did the midwife or doctor do a vaginal examination?

Yes, once or twice

Yes, many times (3-6 times or more) during a short labour (1 to 6 hours)

Yes, many times (7 times or more) during a longer labour (7 hours or more)

No, not at all

Text box for comments

32.  When the midwife or doctor did a vaginal examination, did they:

Yes No Not applicable

explain to you why they thought

this was necessary?

ask your permission to do this?

Text box for comments

33. When you were in labour, did the midwife or doctor ‘break your waters’?

Yes

No

No, waters broke naturally

34.  When the midwife or doctor broke your waters, did they:

Yes No Not applicable

explain to you why they thought

this was necessary?

ask your permission to do this?

Text box for comments

**[You are now just over half way through the survey – thank you! We would be really glad if you would please keep going and answer the next questions as well ☺]**

35. When you were in labour, did the midwife or doctor give you any drugs (e.g., for pain relief, or to speed up labour (usually in a drip), or to speed up birth of the placenta)? Please mark all that apply.

Yes, for pain relief

Yes, to speed up labour

Yes, to speed up birth of the placenta

Yes, other drugs

Don´t know

No

36. When the midwife or doctor gave you a drug, did they:

Yes No Not applicable

explain to you why they thought

this was necessary?

explain all positive and negative impacts

on you or the baby?

ask your permission to do this?

Text box for comments

37. If the midwife or doctor gave you a drug for pain relief, was it:

(please mark all that apply)

An injection

An epidural

Entonox, or ‘gas and air’ that you breathed in

I had no drugs given to me for pain relief

Don’t know

Text box for comments

38. Were you allowed to drink fluids in labour?

Yes

No

Text box for comments

39. Were you allowed to eat light diet in labour (e.g., yoghurt, soup, bread, biscuits, fruit smoothies)?

Yes

No

Text box for comments

40. At any stage in labour, did you refuse any treatment or intervention offered (e.g., to have your waters broken, to have an oxytocin infusion put up (to speed up your labour), to have electronic fetal monitoring applied)

Yes

No

41. Open question: If yes, what was the treatment or intervention, what was the midwife, doctor or doula’s reaction, and what was the result (did you have the treatment or intervention eventually)?

(Please describe in 1-2 sentences)

Text box

42. During your labour, did the midwife or doctor do any of these procedures?

Yes    No    Don’t know

Shave the hair off your perineum

(the area around your vagina)

Give you an enema

Ask questions and take down details from you

while you were having a contraction

Put in an intravenous “drip” to give you fluids

(with no reason)

Push back your cervix (opening of your womb)

when it was nearly, but not completely, open

Offer you drugs to relieve pain

Tie your legs up in stirrups

43. What type of birth did you have?


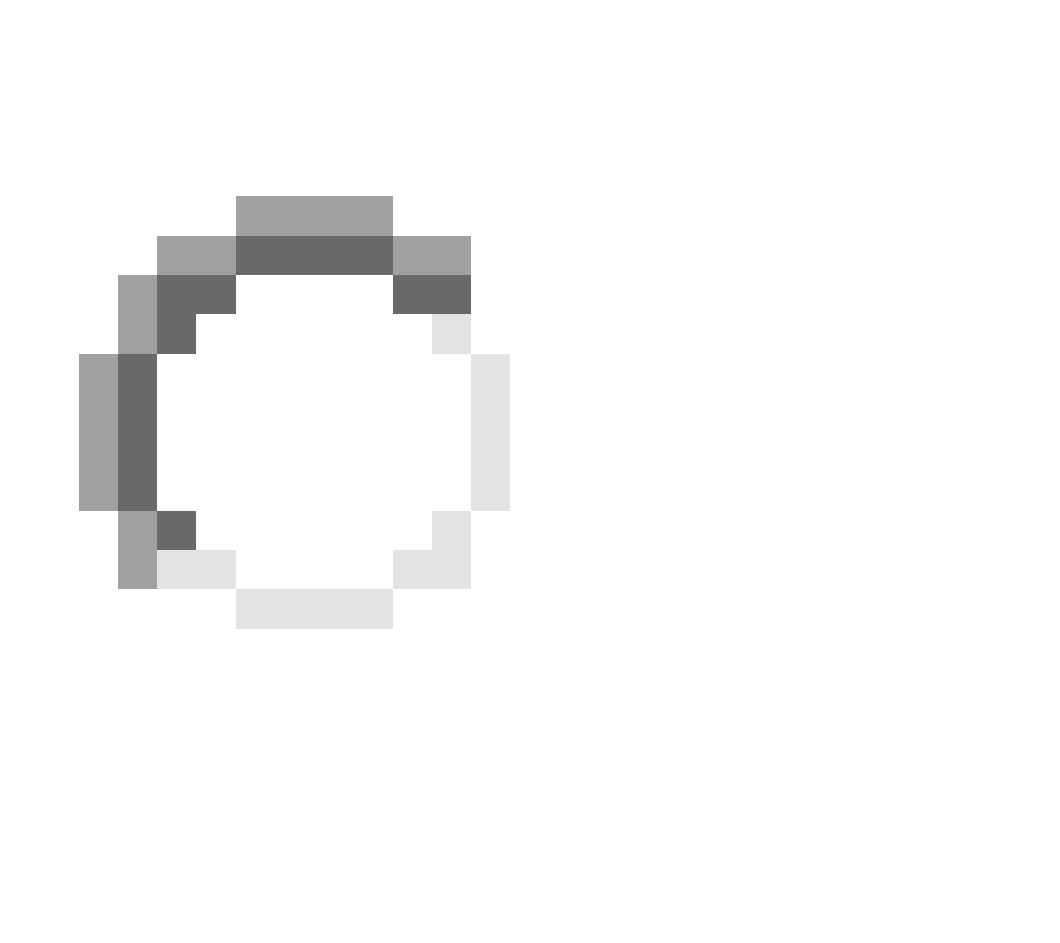
Spontaneous vaginal birth


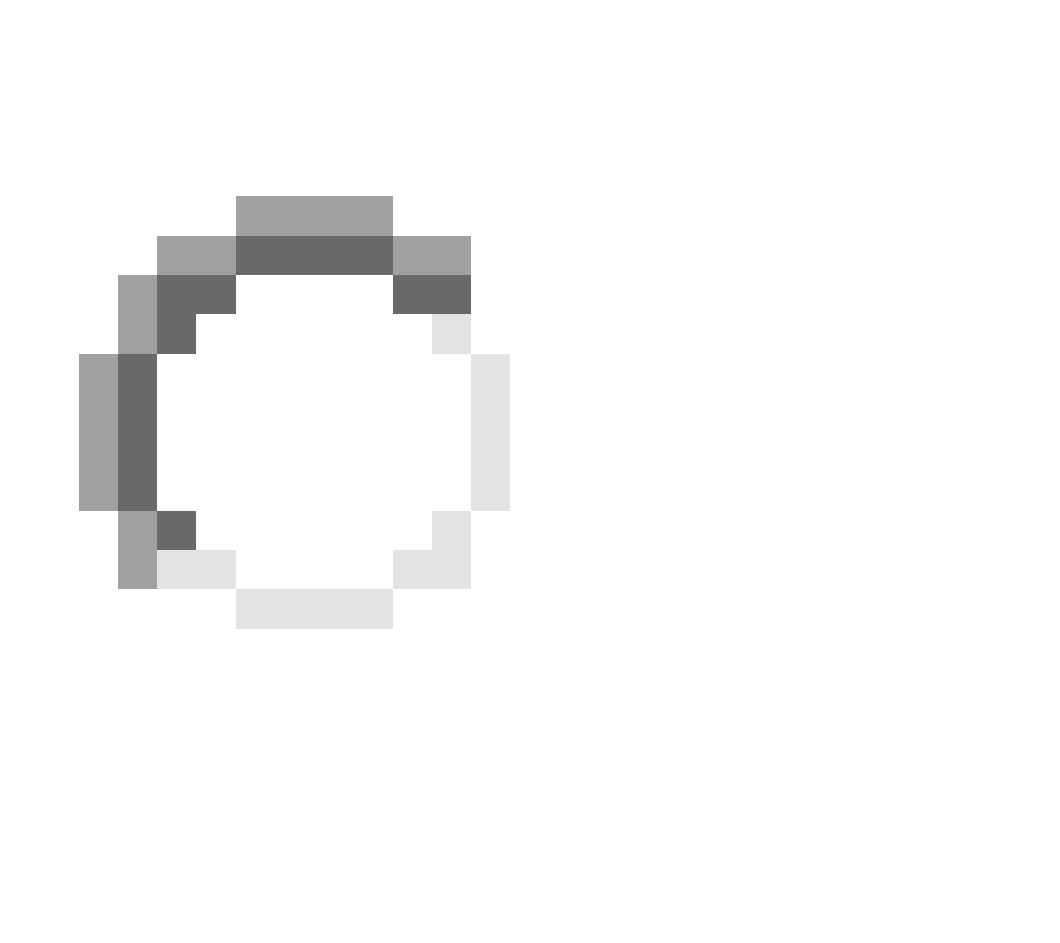
Instrumental birth (Forceps or Vacuum (sometimes referred to as suction))


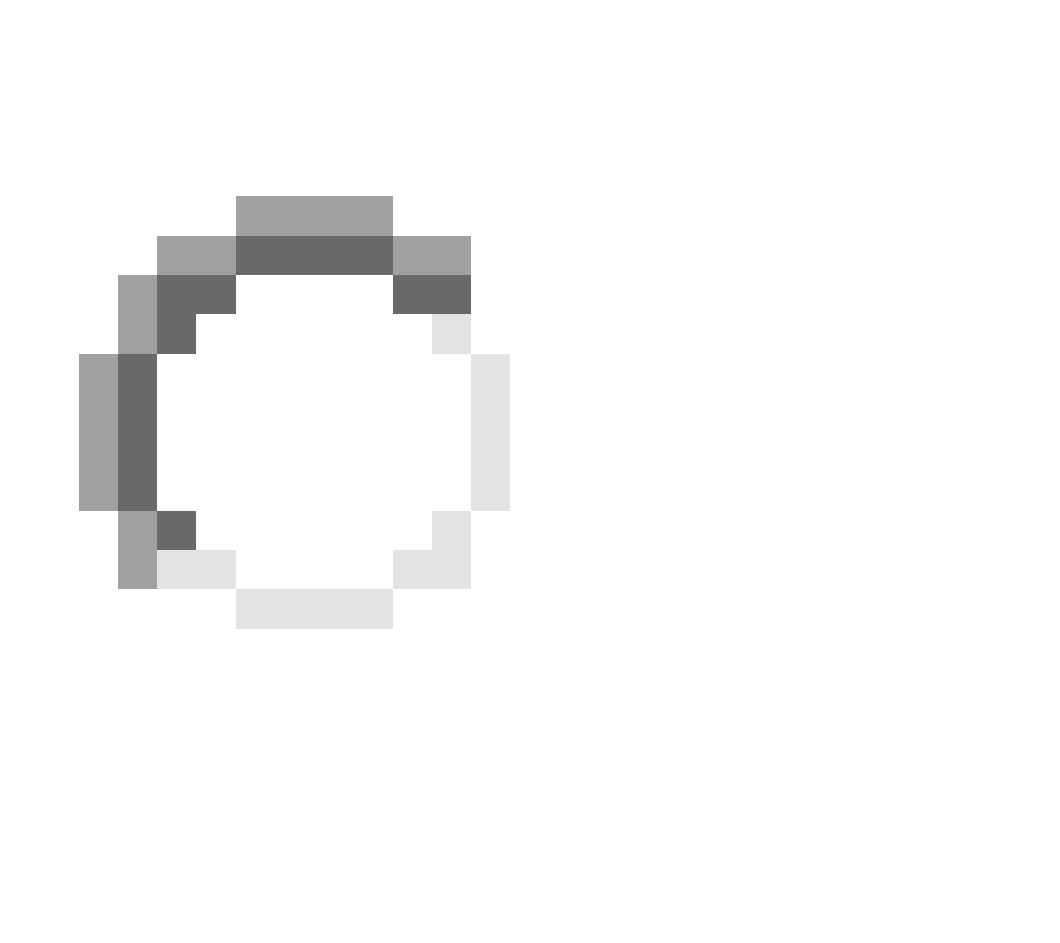
Caesarean section following a period of labour, but not near the birth (please go to question 58)

Caesarean section following a period of labour, and some attempts at pushing or actual birth (please continue to answer the following questions)


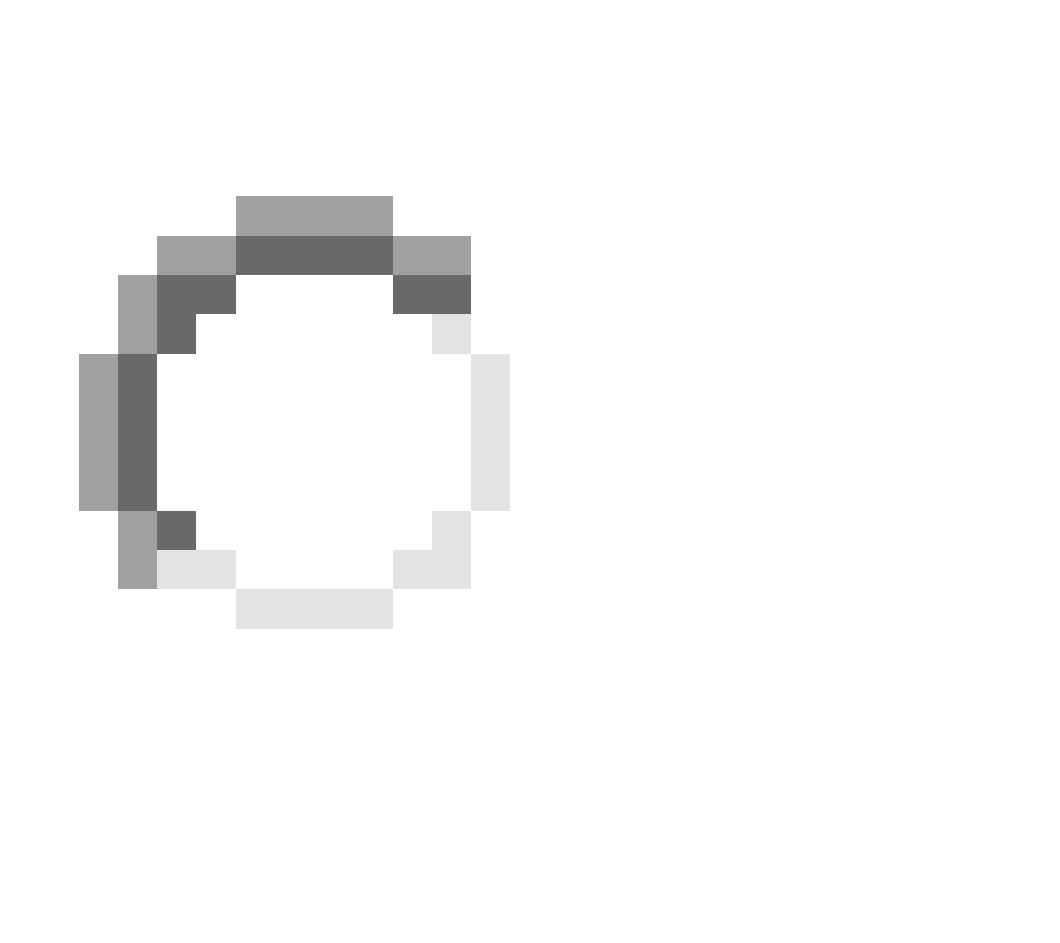
Other (please specify)

Text box for comments

44. If you had a spontaneous vaginal birth, what position were you in for the birth?

Not applicable, had a caesarean section late in labour

Upright (standing, squatting, kneeling, on all fours, on a birthing stool)

Semi-recumbent (on a bed or couch, propped up with a back-rest or a lot of pillows)

Lying flat on a bed or couch, with one or two pillows under your head

Lying on your side on a bed or couch

Water birth

Other, please give details

Text box for comments

45. Were you asked to push as soon as your cervix was fully dilated, before you felt an urge to push?

Yes    No    Don’t know Not applicable

46. Were you encouraged to take a deep breath, hold it, and push down hard for a long time to push your baby out?

Yes    No    Don’t know Not applicable

47. At any stage during the vaginal birth, did the midwife or doctor press hard on your abdomen to push the baby out?

Yes, for a short time (one or two contractions)

Yes, for many contractions

No, not at all

Not applicable

Text box for comments

48.  If, at any stage during the vaginal birth the midwife or doctor pressed hard on your abdomen to push the baby out, did they:

Yes No Not applicable

explain to you why they thought

this was necessary?

ask your permission to do this?

stop if you asked them to?

Text box for comments

49. If, at any stage during the vaginal birth the midwife or doctor pressed hard on your abdomen to push the baby out, how did this feel?:

Text box for comments

50. Did the doctor or midwife assisting you to birth your baby make a cut at the end of your vagina (called an episiotomy) that had to be stitched up afterwards?

Yes

No

Don’t know

Not applicable (had a caesarean section)

51. If the doctor or midwife assisting you to birth your baby made a cut at the end of your vagina (called an episiotomy), did they:

Yes No Not applicable

explain to you why they thought

this was necessary?

ask your permission to do this?

give you a local anaesthetic first to

numb the area?

Text box for comments

52. Did the doctor or midwife assisting you to birth your baby pull on the baby’s head or body immediately it was out, to get him/her born more quickly?

Yes

No

Don’t know

Not applicable

53. If the doctor or midwife assisting you to birth your baby pulled on the baby’s head or body immediately it was out, were you told a reason for this?

Yes

No

Not applicable

Text box for comments

54. Did your baby have any injuries from birth (e.g., severe bruises, fractured bones, nerve damage, paralysis)?

Yes (please specify in box below)

No

Text box for comments

55. Was the baby’s cord left intact for 2-3 minutes after birth, or was it clamped and cut immediately?

Clamped and cut immediately

Left for 2-3 minutes before clamping

Left unclamped for a long time/until the cord stopped pulsating

Don’t know

Text box for comments

**[Just ten more questions to go! Thank you for staying with the survey ☺]**

56. Did you need to have your perineum/vaginal area stitched after the birth?

Yes

No

57. If you did have stitches in your perineum/vaginal area after the birth, did the doctor or midwife use a local anaesthetic first to numb the area?

Yes

No

Don’t know

Not applicable

Text box for comments

**[All the following questions are for you if you had either a vaginal birth or a caesarean section]**

58. After birth/caesarean section was your baby placed skin to skin with you immediately, or very shortly, after birth?


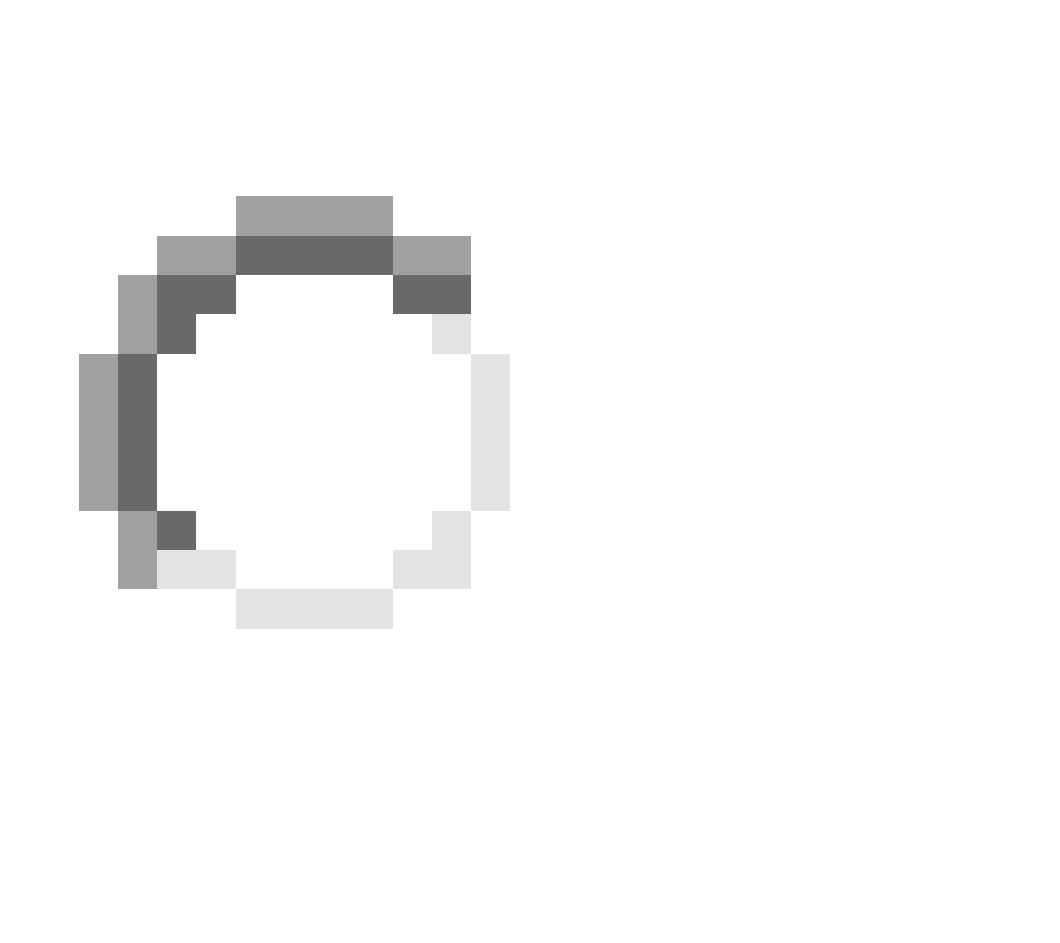
Yes


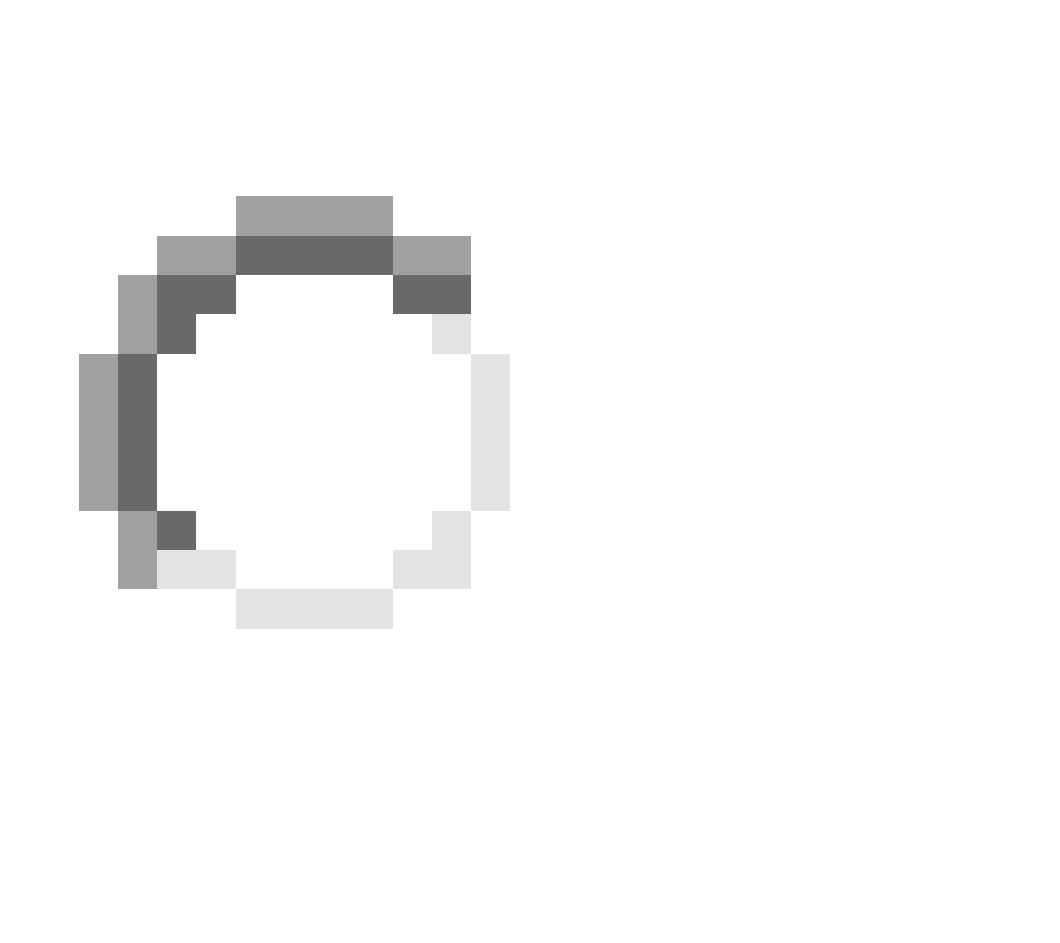
No


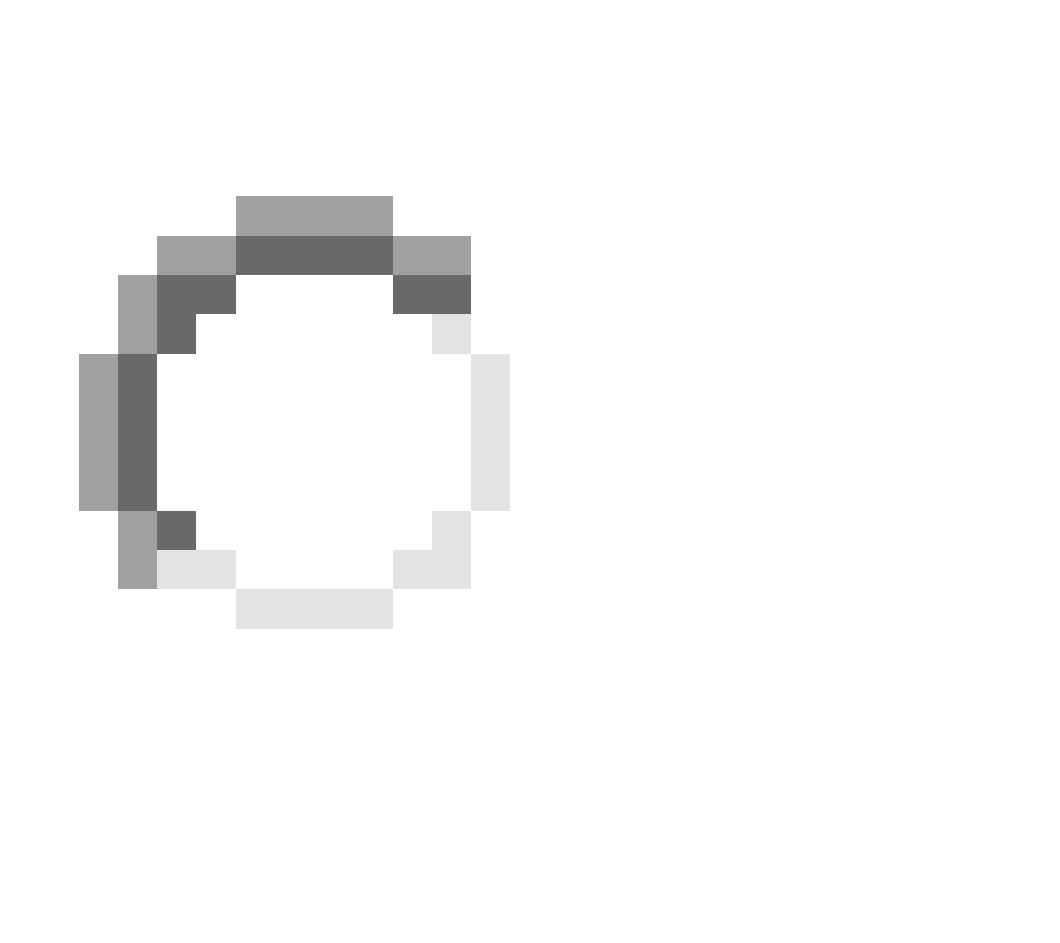
Can't remember


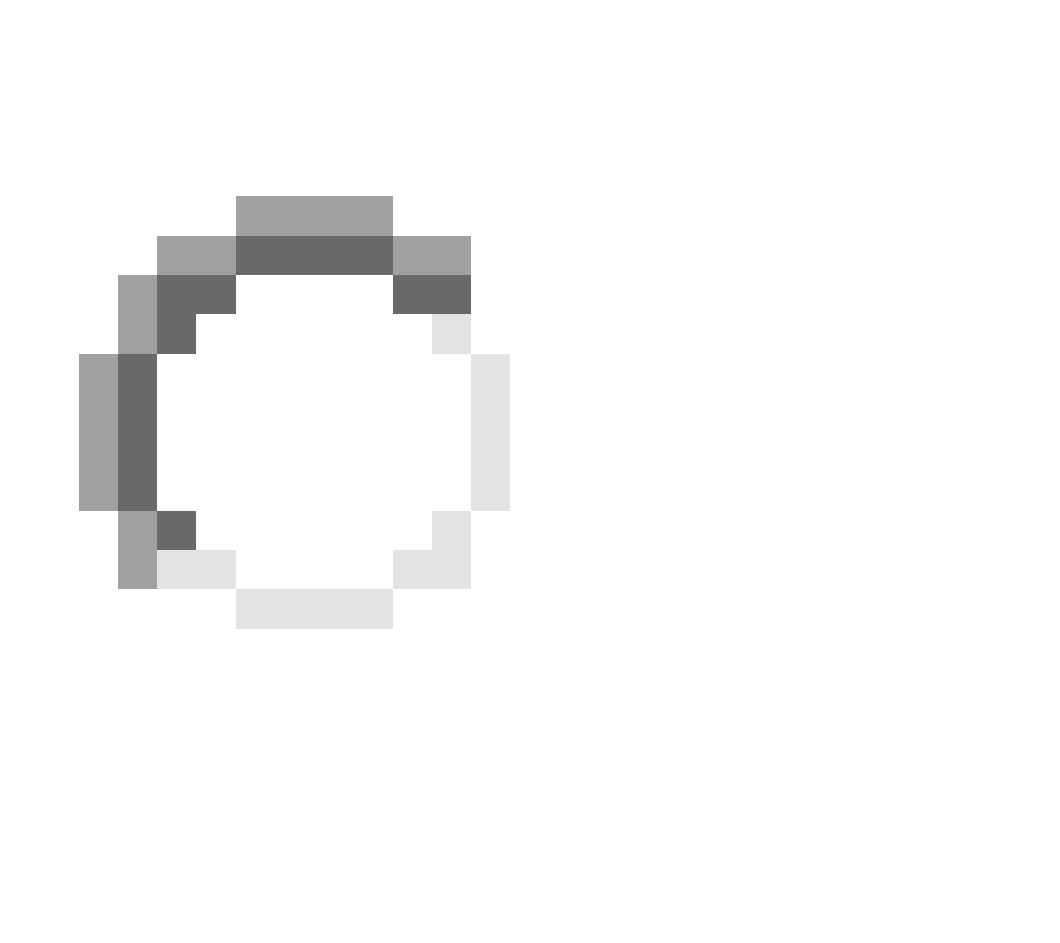
Other (please specify)

Text box for comments

59. Did your baby remain skin to skin with you without any separation for one hour or more?


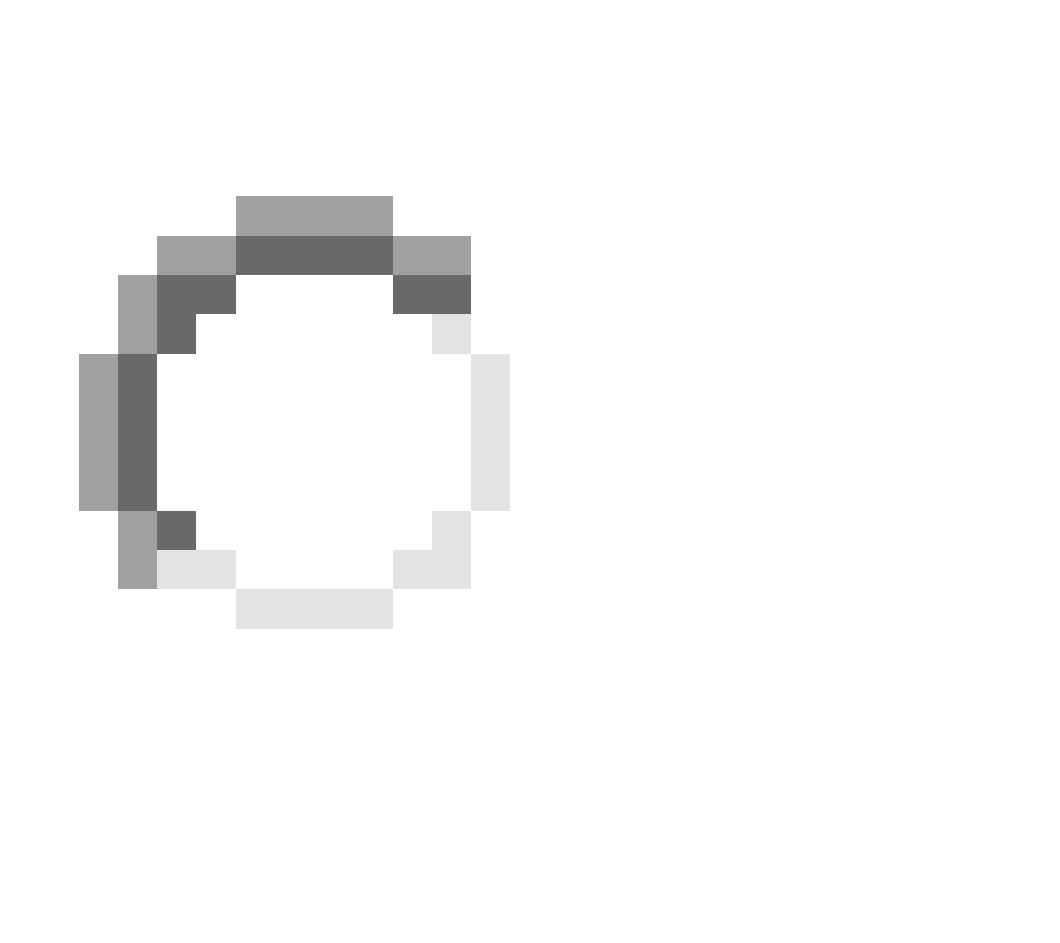
Yes


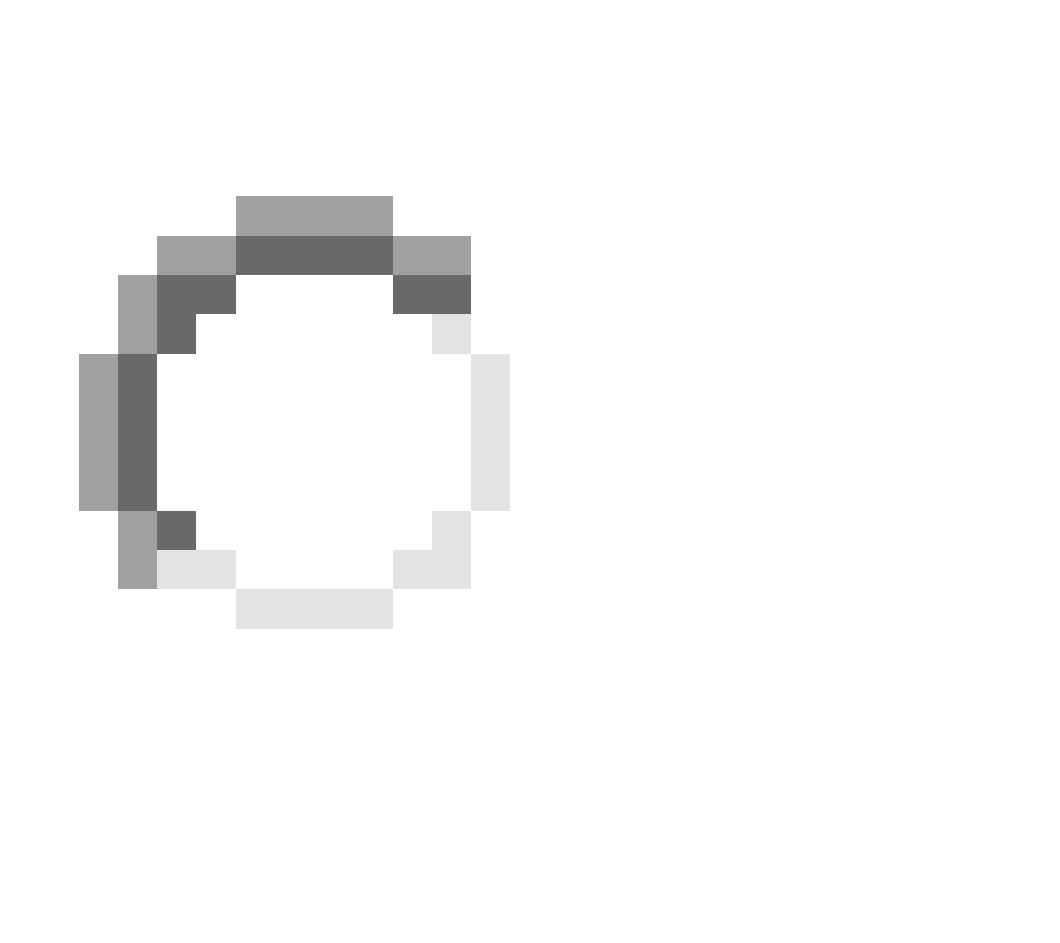
No


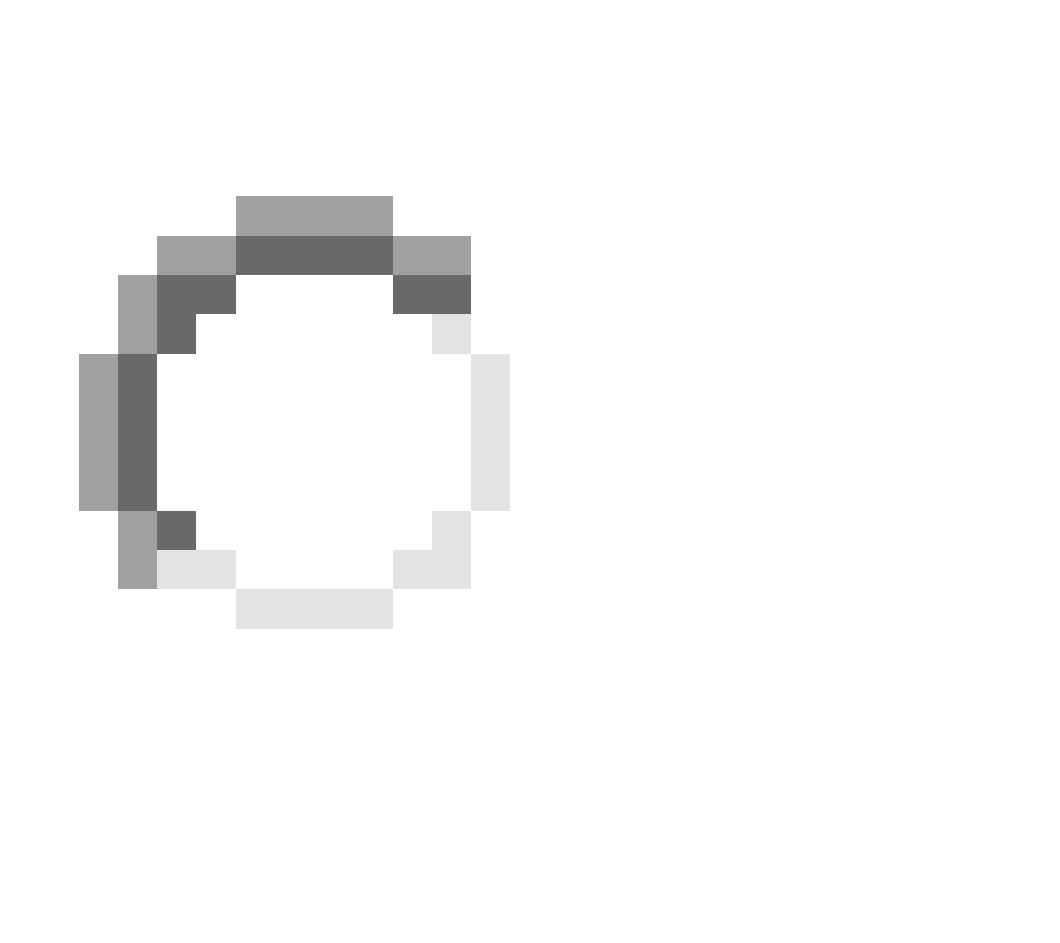
Can't remember


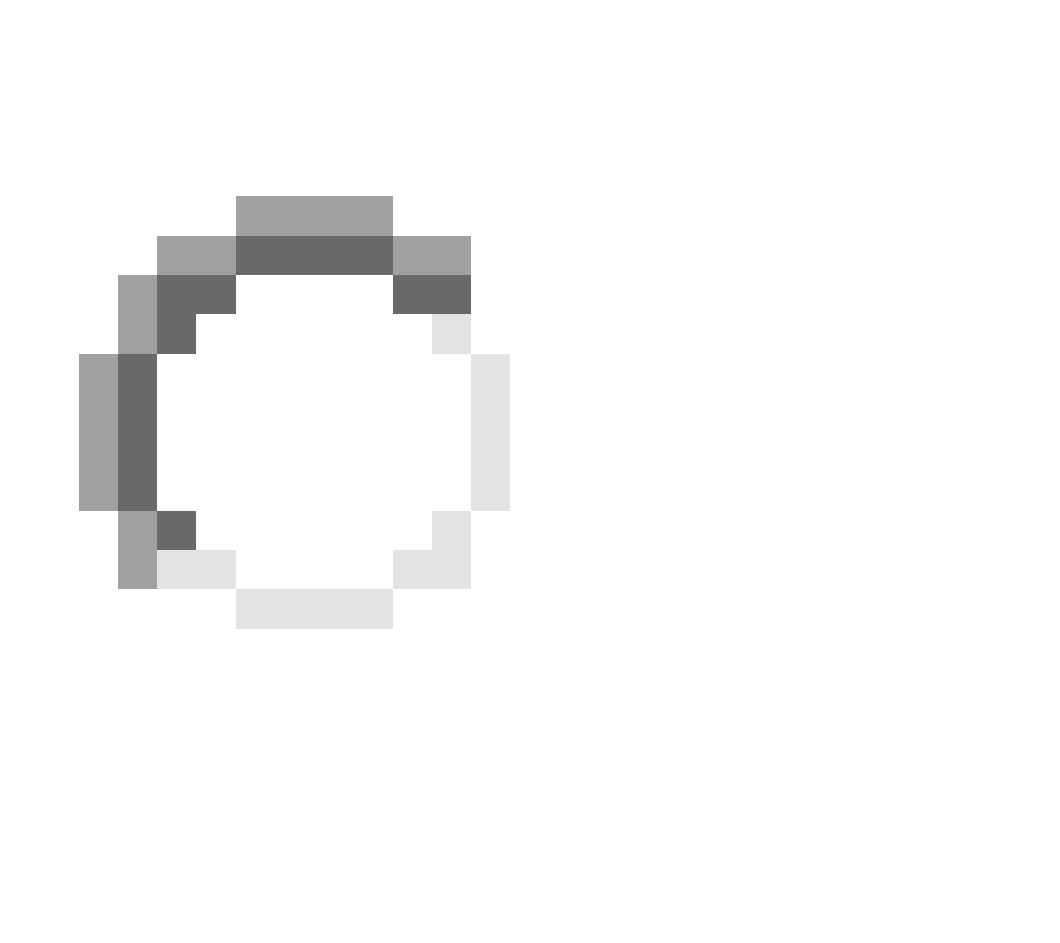
Had no skin-to-skin time at all

Other (please specify)

Text box for comments

60. Did your baby stay beside you at all times while you were in hospital?


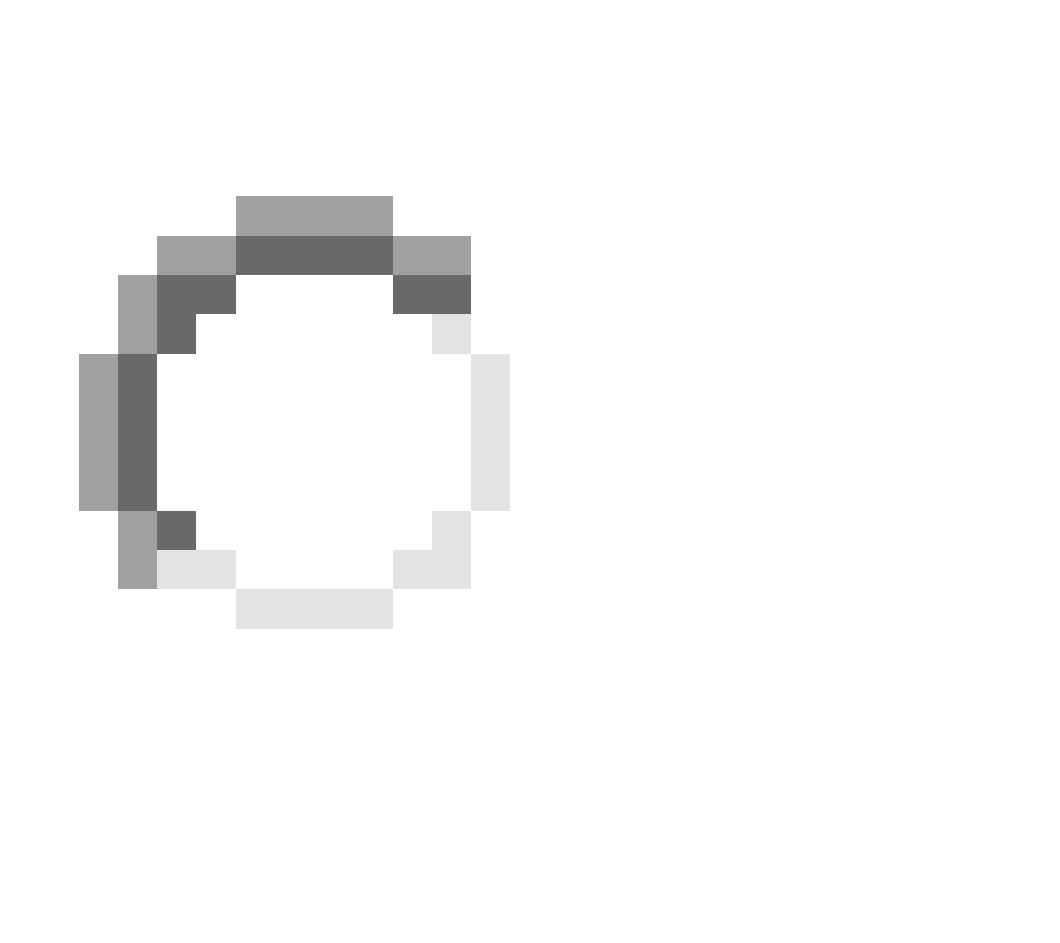
Yes


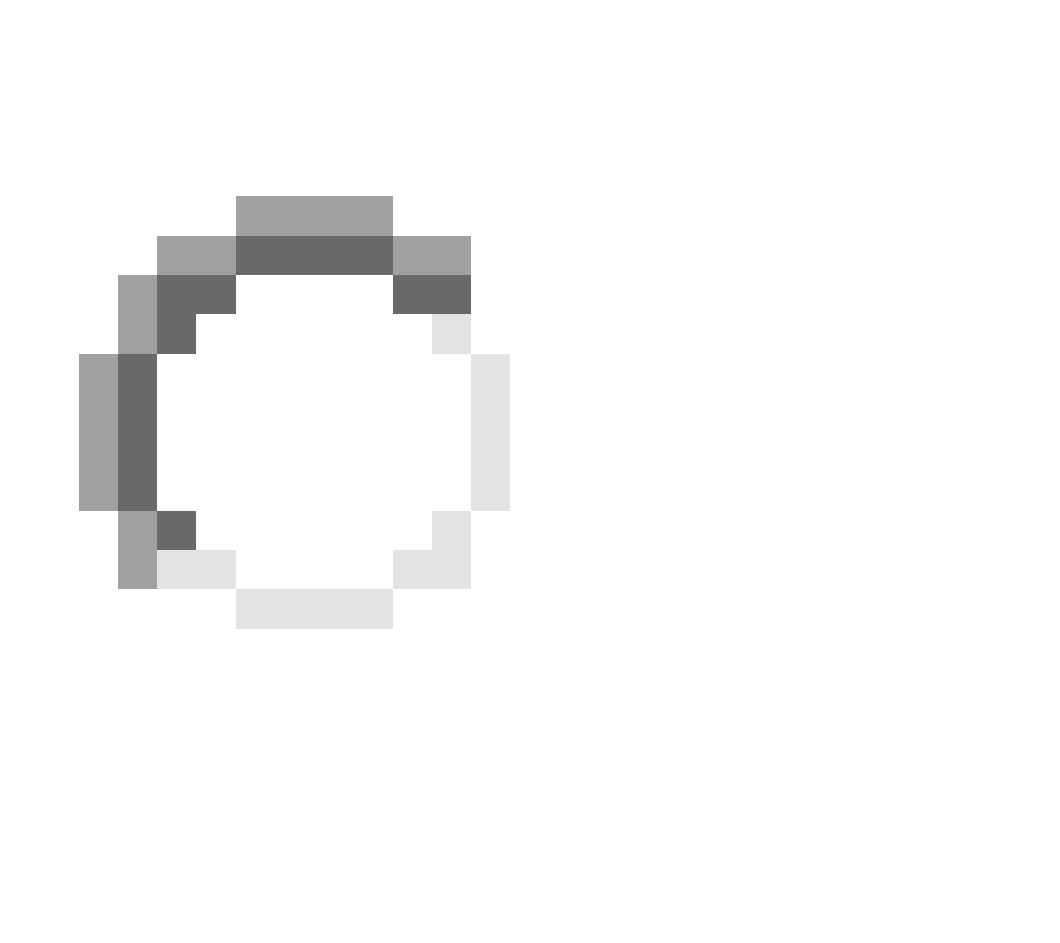
No


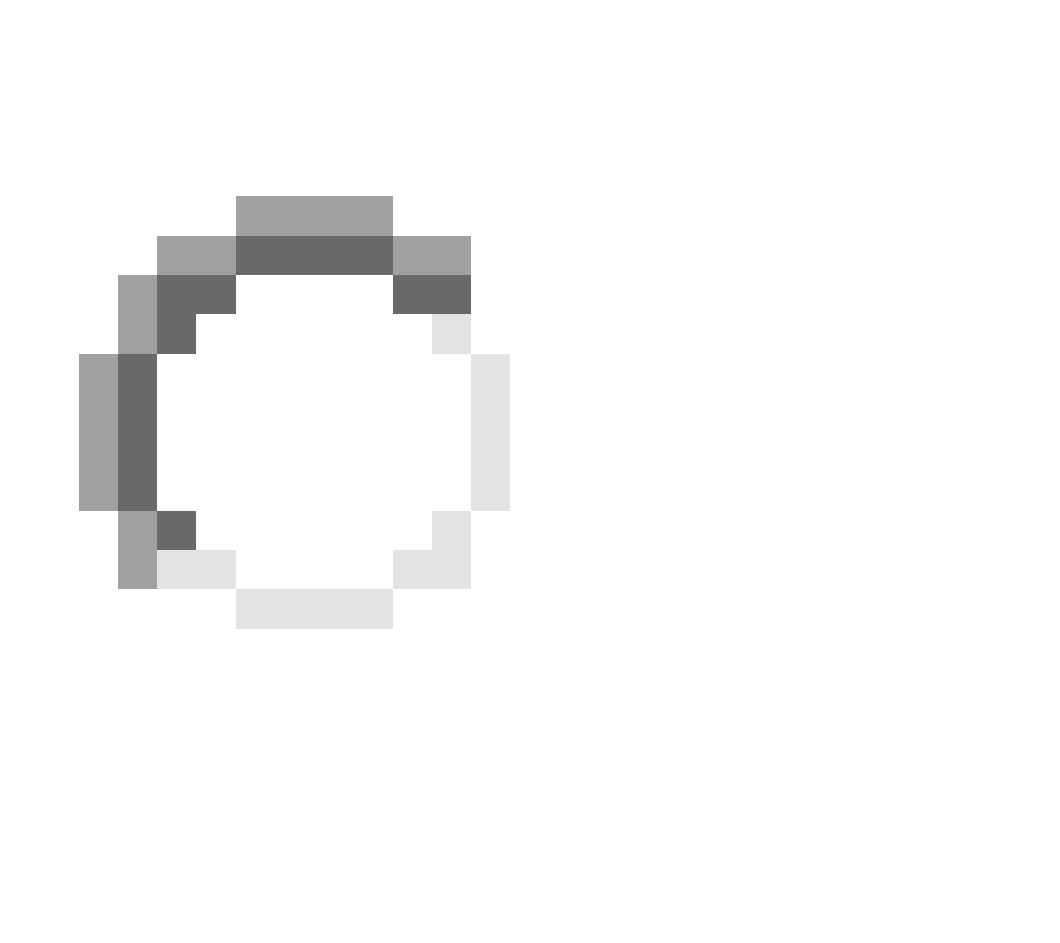
Can't remember


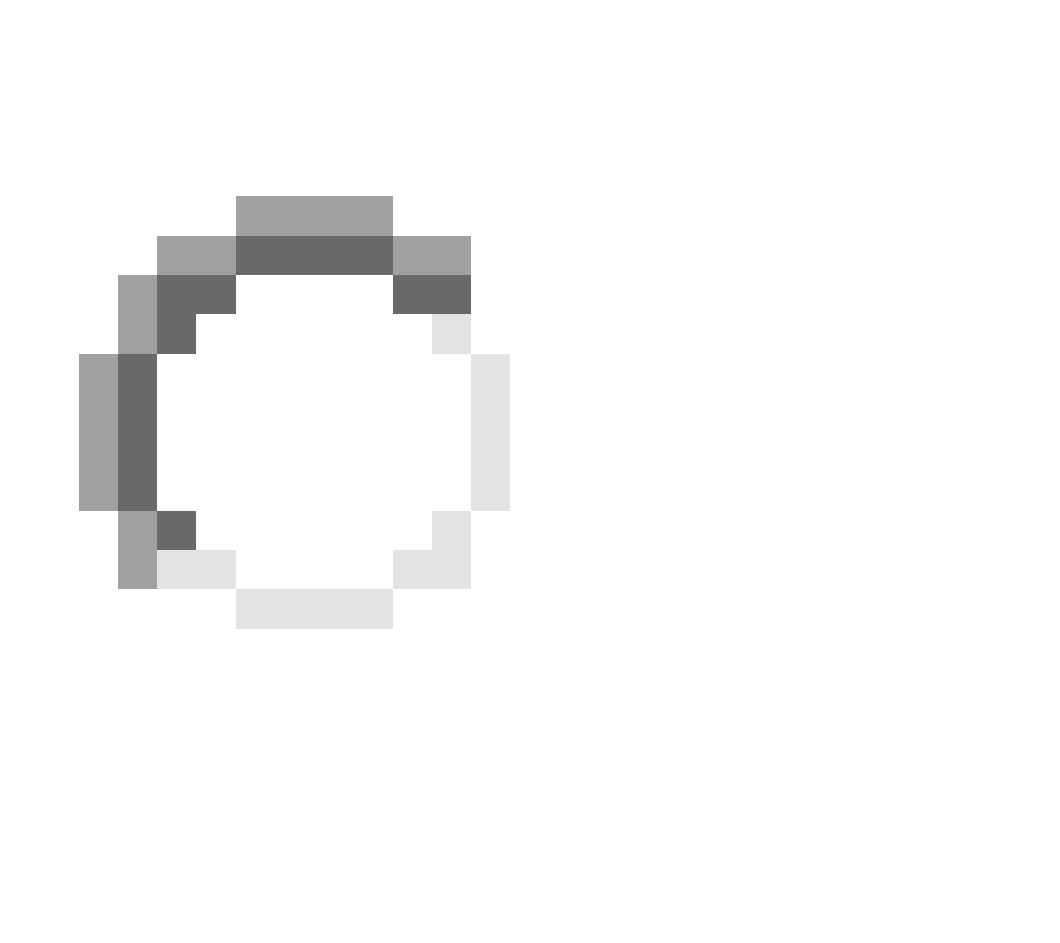
My baby was born at home

Text box for comments

61. Were you, or the father of your baby, present for every examination of your baby?

Yes

No

Don’t know

Text box for comments

62. How long did you stay in hospital after your birth?

I had a home birth

Less than 24 hours (less than 1 day)

Between 24 and 48 hours (more than 1 and less than 2 days)

Between 49 and 72 hours (more than 2 and less than 3 days)

More than 72 hours (more than 3 days)

63. Were you able to decide when you went home from hospital after the birth?

Yes

No

Text box for comments

64. Did you want to leave hospital before the doctor said you could (usually 72h after birth)?”

Yes                              No

If yes, what was the outcome? (You can describe how it was if you like in the comments box)

I was supported by doctors in my decision

I had to argue before they gave me permission to leave

I was too tired/did not feel like arguing so I just stayed in hospital

They did not allow me to leave (Please tell us the reason for the doctors’ decision)

Text box for comments

65. What place of birth would you choose, if you were having another baby and more options were available?

Hospital with intensive care unit

Smaller hospital

Birth centre within/nearby a hospital

Free-standing birth centre (not connected to a hospital)

Home-birth assisted by a qualified midwife

Home-birth assisted by a doula

Home-birth with just my family or friends present

Other

Text box for comments

**Thank you for taking part, and completing all the questions.** We really appreciate your information. Please feel free to add any extra comments if you wish.

Text box for comments

**If you would like support or advice for any of these issues, this is available from** [**http://apodac.org/index.php/apodac/**](http://apodac.org/index.php/apodac/)
